# Human Chromosome 18 and Acrocentrics: A Dangerous Liaison

**DOI:** 10.3390/ijms22115637

**Published:** 2021-05-26

**Authors:** Nicoletta Villa, Serena Redaelli, Elena Sala, Donatella Conconi, Lorenza Romitti, Emanuela Manfredini, Francesca Crosti, Gaia Roversi, Marialuisa Lavitrano, Ornella Rodeschini, Maria Paola Recalcati, Rocco Piazza, Leda Dalprà, Paola Riva, Angela Bentivegna

**Affiliations:** 1 Medical Genetics Laboratory, Clinical Pathology Department, S. Gerardo Hospital, 20900 Monza, Italy; n.villa@asst-monza.it (N.V.); elena.sala@asst-monza.it (E.S.); f.crosti@asst-monza.it (F.C.); 2 School of Medicine and Surgery, University of Milano-Bicocca, 20900 Monza, Italy; serena.redaelli@unimib.it (S.R.); gaia.roversi@unimib.it (G.R.); marialuisa.lavitrano@unimib.it (M.L.); rocco.piazza@unimib.it (R.P.); leda.dalpra@unimib.it (L.D.); 3 Pathology and Cytogenetics Laboratory, Clinical Pathology Department, Niguarda Ca’ Granda Hospital, 20162 Milan, Italy; lorenza.romitti@ospedaleniguarda.it; 4 Medical Cytogenetics Laboratory, Istituto Auxologico Italiano IRCCS, 20095 Cusano Milanino, Italy; e.manfredini@auxologico.it (E.M.); rodeschini.o@alice.it (O.R.); p.recalcati@auxologico.it (M.P.R.); 5 Medical Genetics Laboratory, Medical Biotechnology and Translational Medicine Department, University of Milan, 20090 Milan, Italy; paola.riva@unimi.it

**Keywords:** acrocentric chromosomes, chromosome 18, centromeric/pericentromeric regions, chromosome translocations, chromosome territory, nuclear subcompartments

## Abstract

The presence of thousands of repetitive sequences makes the centromere a fragile region subject to breakage. In this study we collected 31 cases of rearrangements of chromosome 18, of which 16 involved an acrocentric chromosome, during genetic screening done in three centers. We noticed a significant enrichment of reciprocal translocations between the centromere of chromosome 18 and the centromeric or pericentromeric regions of the acrocentrics. We describe five cases with translocation between chromosome 18 and an acrocentric chromosome, and one case involving the common telomere regions of chromosomes 18p and 22p. In addition, we bring evidence to support the hypothesis that chromosome 18 preferentially recombines with acrocentrics: (i) the presence on 18p11.21 of segmental duplications highly homologous to acrocentrics, that can justify a NAHR mechanism; (ii) the observation by 2D-FISH of the behavior of the centromeric regions of 18 respect to the centromeric regions of acrocentrics in the nuclei of normal subjects; (iii) the contact analysis among these regions on published Hi-C data from the human lymphoblastoid cell line (GM12878).

## 1. Introduction

Centromeres represent the chromosomal domains required to ensure faithful transmission of the genome during cell division. In addition to inducing numerical chromosome alterations, centromere dysfunctions could also destabilize chromosome integrity, leading to structural alterations. This intrinsic fragility is probably due to the high density of repetitive sequences that makes the centromere more vulnerable and prone to rearrangements [1]. Centromeres may break and rearrange with other centromeres or pericentromeric regions forming dicentric or pseudodicentric derivatives (with inactivation of one/two centromeres) or creating a new centromere by a fusion event (centric fission) [2]. In particular, Robertsonian translocations, that originate from whole arm exchanges involving only acrocentric chromosomes (13, 14, 15, 21, 22), result in a dicentric or pseudodicentric chromosome, with inactivation of one centromere [3]. As a matter of fact, Robertsonian translocations are the most common human chromosomal rearrangement (1 in 1000 live births), with a de novo mutation rate of 0.011% [4,5]. On the other hand, among the non-acrocentric ones, chromosome 18 is one of the most involved in structural rearrangements. Reciprocal translocations, both familiar and de novo, isochromosomes p and q, duplications or deletions, were reported; many cases of 18p- deletion have also been described [6]. In addition, patients with 18p- Syndrome (incidence of 1:50,000) and a variable but well-defined clinical picture [7,8,9], most frequently show breakpoints in the pericentromeric region, in close proximity to the centromere. Another peculiarity is that acrocentric chromosomes are preferentially involved in chromosome 18 rearrangements (16% in [6]).

We collected 31 cases of rearrangements of chromosome 18, of which 16 involved an acrocentric chromosome. Here, we describe five cases with translocation between the centromere of chromosome 18 and the centromeric or pericentromeric regions of the acrocentrics: one with chromosome 13, two with chromosome 22, another one with chromosome 15 and the last one with chromosome 21. In addition, we report one case involving the common telomere regions of chromosomes 18p and 22p. Bioinformatic analysis done on Hi-C data generated from the human lymphoblastoid (GM12878) cell line to evaluate the relationships between centromeric/pericentromeric regions of chromosome 18 and acrocentric chromosomes support these preferential interchromosomal interactions, also observed by 2D-FISH analysis on nuclei of phenotypically normal subjects, and suggests that chromosome 18 behaves as if it were an acrocentric one. 

## 2. Case Reports

### 2.1. Case 1

#### 2.1.1. Case Description

A 43-year-old woman requested prenatal diagnosis on amniotic fluid at 16^+5^ weeks of gestation. After genetic counselling for the cytogenetic and molecular finds (see below) the couple decided to continue pregnancy that was complicated by gestational diabetes and spontaneously ended at 36 weeks. At birth, the Apgar score was 8/9, birth weigh 2104 g (15th percentile), length 49 cm (88th percentile), and head circumference 31 cm (15th percentile). The neuropsychiatric evaluations at birth, 13, and 30 months showed a regular psychomotor development with normal socialization. No dysmorphic features and physical abnormalities were observed at clinical examination.

#### 2.1.2. Results

**Figure 1:**
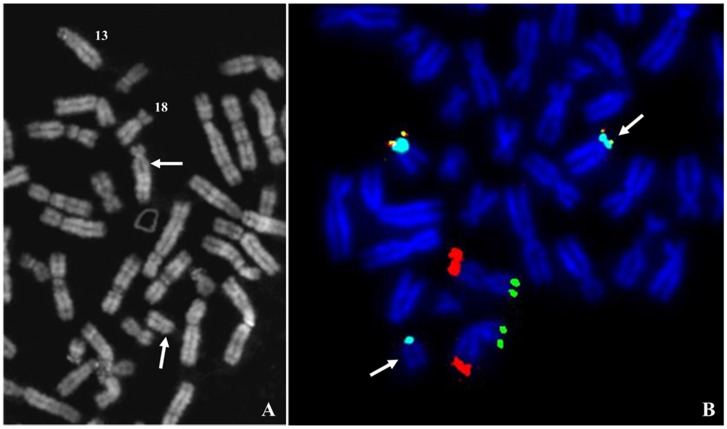
Case 1. (**A**) Partial Q banded metaphase plate, the arrows indicate the two chromosomes involved in the balanced translocation t(13;18)(q10;q10), the numbers indicate the normal homologs. (**B**) Metaphase plate after FISH with mixture of telomeres specific for chromosomes 11 and 18p. The arrows indicate the two derivatives of translocation. Signals: green 11ptel; red 11qtel; yellow 18ptel; aqua D18Z1.

The cytogenetic analysis on 14 colonies derived from amniotic fluid cells showed an apparently deleted 18p and a chromosome 13 with elongated p arm and no satellites (Figure 1A). FISH analysis with subtelomeric probes showed the presence of 18p subtelomeres on one chromosome 13, together with the signal for the centromeric region of chromosome 18. A small centromeric signal was also present on the derivative chromosome 18, apparently deleted, confirming a translocation involving the p arms of this two chromosomes. Normal signals were observed on subtelomeric regions of chromosomes 11 available in the same mixture of used probes (Figure 1B). The parents’ karyotypes were normal, with no informative chromosome polymorphisms, defining a de novo origin of the rearrangement observed. The karyotype of the proband was:

46,XX,der(13)(18pter→18p11.1::13p11.1→13qter),der(18)(18p11.1→18qter)dn.

ish der(13)(D18S552+,D18Z1),der(18)(D18S552-,D18Z1+)

Array-CGH analysis showed no copy-number alteration on the involved chromosomes, therefore confirming the balanced nature of the rearrangement. Of note, a duplication of 246 kb (from nt 144853176 to 145099318) in Xq27.3, maternally inherited, involving *SLITRK2* and *TMEM257* genes, was identified as an incidental finding.

### 2.2. Case 2

#### 2.2.1. Case Description

A 34 year-old male patient came to the attention of medical genetics laboratory for a FISH analysis of a de novo unbalanced translocation der(18)t(18;22), diagnosed in another center.

The patient was born by a cesarean section due to arrested labor and fetal distress and was subjected to resuscitation. At birth, he presented feeding difficulties, milk allergy and poor growth. Autonomous walk and language development were achieved at 2- and 3-years old respectively. At 9-years old, an encephalic TAC showed a modest enlargement of the ventricular system. The current clinical picture is characterized by psychomotor delay, behavioral problems, muscular hypotonia, walking ataxia, and polycythaemia vera.

#### 2.2.2. Results

**Figure 2:**
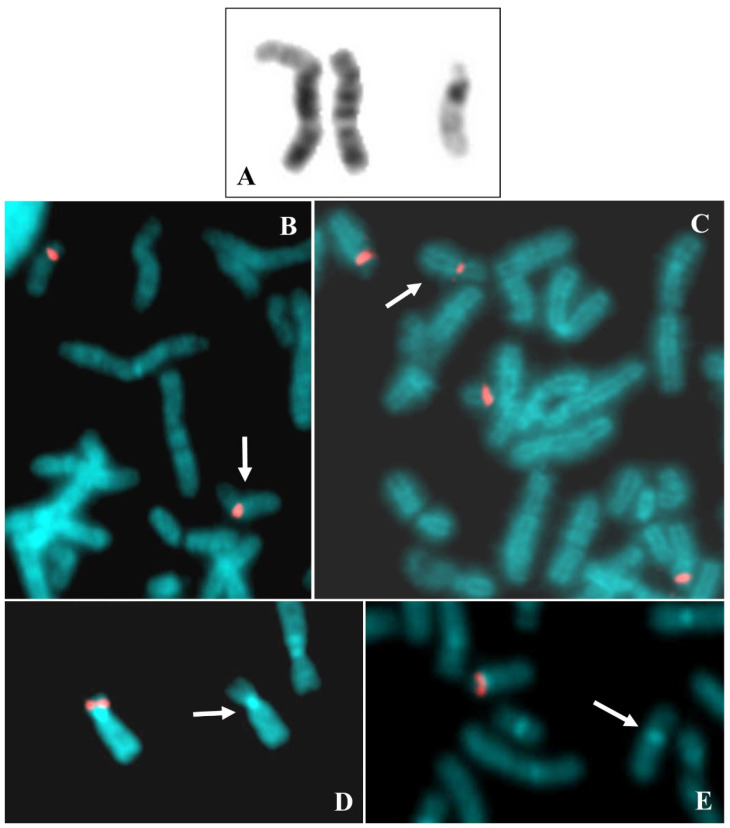
Case 2. (**A**) Chromosomes 18 and 22 from a GTG stained metaphase, left chromosome 18 translocated with 22q, right normal chromosomes 18 and the normal 22. (**B**) Partial metaphase after FISH with D18Z1, the arrow indicates t(18;22)(q10;q10). (**C**) FISH with probe D14Z1D22Z1, the arrow indicates t(18;22)(q10;q10). (**D**) FISH with BAC RP11 19M2 (18p 11.21), the arrow indicates t(18;22)(q10;q10). (**E**) FISH with BAC RP11 48H7 (18p 11.21), the arrow indicates t(18;22)(q10;q10).

The cytogenetic analysis evidenced the loss of one chromosome 22 and the presence of one derivative chromosome composed of 18q and 22q (Figure 2A). D18Z1 centromeric probe hybridized on both chromosomes 18 and D14Z1/D22Z1 common centromeric probe hybridized on both chromosomes 14, on normal chromosome 22 and on the abnormal 18 (Figure 2B,C). FISH analysis with RP11-19M2 and RP11-48H7 probes showed the presence of both signals on normal chromosome 18 and the lack on the abnormal one (Figure 2D,E and Figure S1 for probes mapping).

Taken together, these data revealed that both breaks occurred at centromere, with the exchange of whole q-arms and the loss of acentric p-arms. Therefore, the karyotype of the proband was:

45,XY,der(18;22)(q10;q10)dn.ish der(18;22)(RP11-19M12-,RP11-48H7-, D18Z1+,

D14Z1/D22Z1+).

The parents’ karyotypes were normal. The paternal origin of derivative 18 was based both on the study of chromosome 22′s polymorphisms and on microsatellites analysis. Five microsatellites mapping on 18p arm were checked: three were not informative (D18S59, D18S464, D18S63) and two showed only the maternal allele (D18S1140 and D18S53) (data not shown; Figure S1 for probes mapping).

### 2.3. Case 3

#### 2.3.1. Case Description

The karyotype for a two-month-old child with labiopalatoschisis, holoprosencephaly, and neonatal diabetes was requested. At the age of 4, the follow-up revealed postural defects, lack of language, and severe mental retardation.

#### 2.3.2. Results

**Figure 3:**
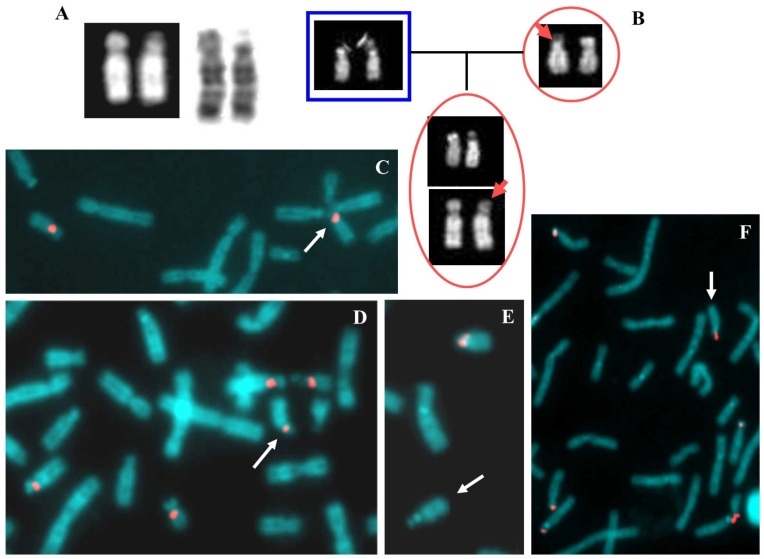
Case 3. (**A**) Chromosomes 18, QFQ (**left**) and GTG (**right**) banded, the 18ps is on the right of the couple. (**B**) Family tree showing the paternal chromosomes 22 on the left, those maternal on the right, and the daughter with 18ps together with 22 on the bottom. The arrows indicate the 22 maternal chromosome probably involved in the translocation. (**C**) Partial metaphase after FISH with probe D18Z1, the arrow indicates the 18ps in association with other acrocentric chromosomes of D and G groups. (**D**) Partial metaphase after FISH with probe D14Z1D22Z1, the arrow indicates the 18ps. (**E**) Normal chromosome 18 showing hybridization after FISH with probe RP11-48H7 (18p11.21) and the arrowed 18ps without signal of hybridization. (**F**) Partial metaphase after FISH with beta satellite probe, the arrow indicates the 18ps showing signal of hybridization.

The cytogenetic analysis showed a satellite chromosome 18 with an apparent loss of 18p (Figure 3A). The centromeric probes D18Z1, D13Z1/D21Z1, and D14Z1/D22Z1 were used to verify the chromosomes’ correct number and localization. Centromeres of chromosome 18 were in regular position in both homologous; instead, centromere of chromosome 22 was also present on abnormal chromosome 18 (Figure 3C,D). Centromeres of chromosomes 13 and 21 showed a correct number and position (data not shown). The BAC probe RP11-48H7 was not present on the derivative chromosome, confirming the 18p- (Figure 3E). Moreover, TUPLE-1 (22q11.2), ARSA (22q13.3), and whole chromosome painting (WCP) 14 probes were not evident on this chromosome (data not shown), but the presence of cytological satellites was confirmed after FISH with beta-satellite probe (Figure 3F).

Therefore, the karyotype of the proband was:

46,XX,der(18)t(18;?)(p10;?)dn.ish der(18;?22)(q10;p10),+22(RP11-48H7,bet

sat+,D13Z1/D21Z1-,D14Z1/D22Z1+,WCP14-,TUPLE1-,D18Z1+).

The parents’ karyotypes were normal. The maternal origin of 18p- was based both on the study of polymorphisms of chromosomes 14 and 22 (see the family tree in Figure 3B) and on microsatellites analysis. Five microsatellites mapping on 18p arm were checked: three were not informative (D18S53, D18S464, D18S63) and two showed only the paternal allele (D18S1140 and D18S59) (data not shown; Figure S1 for probes mapping).

### 2.4. Case 4

#### 2.4.1. Case Description

A prenatal diagnosis on amniotic fluid (17^+4^ weeks of gestation) was requested for advanced maternal age (39 years). Family history was negative for genetic pathologies. Normal obstetric ultrasound for fetal morphology, intrauterine growth, and at term trouble-free birth were reported.

Currently, at the age of 1 year, an autism spectrum disorder is suspected. No other pediatric problems, major malformations or facial dysmorphisms are referred.

#### 2.4.2. Results

**Figure 4:**
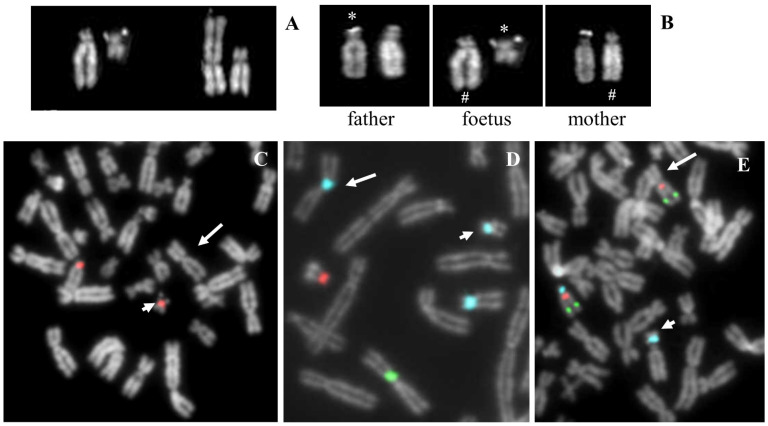
Case 4. (**A**) QFQ banded chromosomes 15 and 18 of the proband. From left to right: normal 15, derivative 15, derivative 18 and normal 18. (**B**) QFQ banded chromosomes 15 of the father (**left**), the foetus (**middle**) and the mother (**right**). #: indicates the normal chromosome 15; *: indicates the satellites of the paternal chromosome. (**C**) FISH with probe D15Z4, centromeric, alpha satellite, the arrow indicates the derivative 18 without signal of hybridization. (**D**) Multicolour FISH with probes D18Z1 (light blue) DXZ1 (green) DYZ3 (red). The arrow indicates the derivative 18 and the arrowhead indicates the derivative 15. (**E**) Multicolour FISH with probes D15Z1 (15p11.2, satellite III, light blue), *SNRPN* (15q11.2, red), *PML* (15q24, green). The arrow indicates the derivative 18 and the arrowhead indicates the derivative 15.

A reciprocal translocation between chromosome 15 and 18 was observed in all the analysed amniocytes (Figure 4A). FISH analysis showed signals for D15Z4 alphoid centromeric probe on both normal and derivative chromosome 15, suggesting that the breakpoint was pericentromeric (Figure 4C); the D18Z1 alphoid centromeric probe also hybridized on both normal and derivative 18, indicating the involvement of chromosome 18′s centromere (p10) in the breakpoint (Figure 4D). Multicolour FISH with D15Z1 (15p11.2), *SNRPN* (15q11.2) and *PML* (15q24) probes revealed that the breakpoint of chromosome 15 was between the centromeric region and the *SNRPN* gene (Figure 4E). Being a prenatal diagnosis and involving a chromosome 15, the methylation test for Prader-Willi/Angelman syndromes was performed, with a negative result.

Moreover, the molecular karyotyping was carried out, with a normal result: arr(X,Y)x1,(1-22)x2, indicating that no genetic material was lost. Polymorphisms analysis of the satellites demonstrated the paternal origin of chromosome 15 involved in the translocation (Figure 4B). Therefore, it is highly probable that the event occurred in meiosis and that chromosome 18 was paternally inherited. The karyotype of the proband was:

46,XY,t(15;18)(q11.2;p10)dn.ish t(15;18)(q11.2;p10)(D15Z1+,D15Z4+,D18Z1+,SNRPN-,PML-;D18Z1+,SNRPN+,PML+).arr (X,Y)x1,(1-22)x2.

### 2.5. Case 5

#### 2.5.1. Case Description

A prenatal diagnosis on amniotic fluid (16th week of gestation) was requested for advanced maternal age (37 years). The pregnancy was interrupted because of a cytogenetic diagnosis of trisomy 18. A subsequent pregnancy was normal and a female was delivered.

#### 2.5.2. Results

**Figure 5:**
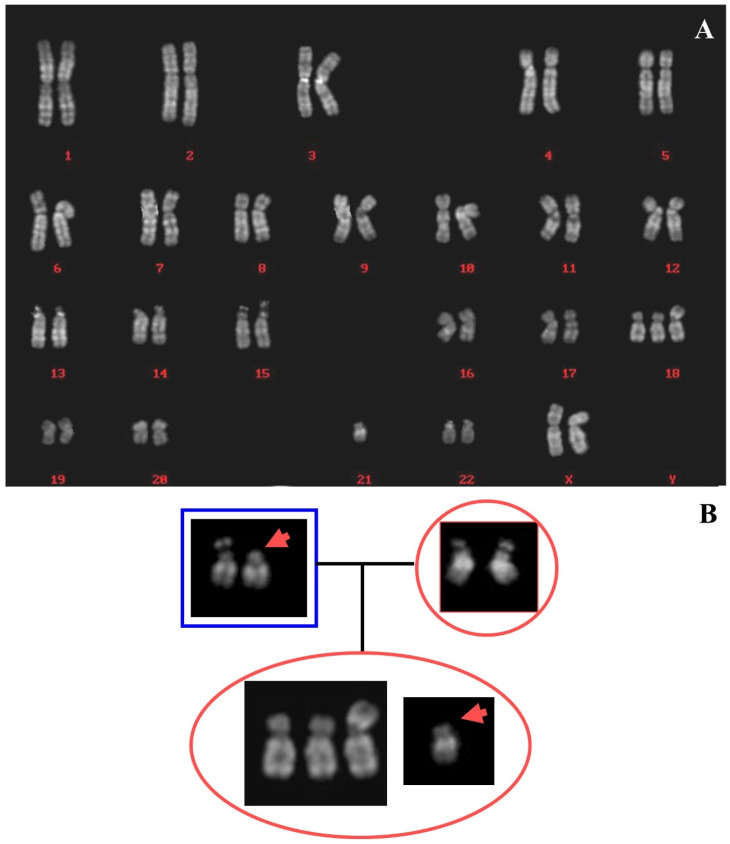
Case 5. (**A**) Reconstruction of karyotype in QFQ bands. (**B**) Family tree showing paternal (**left**), maternal (**right**), and foetus chromosomes 21. The arrowhead indicates the paternal chromosome 21, apparently without satellites.

All the metaphases obtained from amniotic fluid cultures showed the presence of 18q trisomy, due to an unbalanced translocation between chromosomes 18 and 21, with the loss of 18p arm. The parents’ karyotypes were normal, while the proband one was: 46,XX,+18,der(18;21)(q10;q10)dn. (Figure 5A). Polymorphisms analysis of chromosomes 21 suggested the maternal origin of rearranged 21. The normal chromosome 21 seemed to be the paternal satellited one (Figure 5B). Further investigations were not possible for the absence of parents’ consent.

### 2.6. Telomeric Case (Case 6)

#### 2.6.1. Case Description

A 38-year-old man came to the attention of medical genetics laboratory for infertility due to azoospermia/oligospermia. His sister had two children. Family histories of the couple were negative for infertility and abortion and for genetic pathologies.

#### 2.6.2. Results

**Figure 6:**
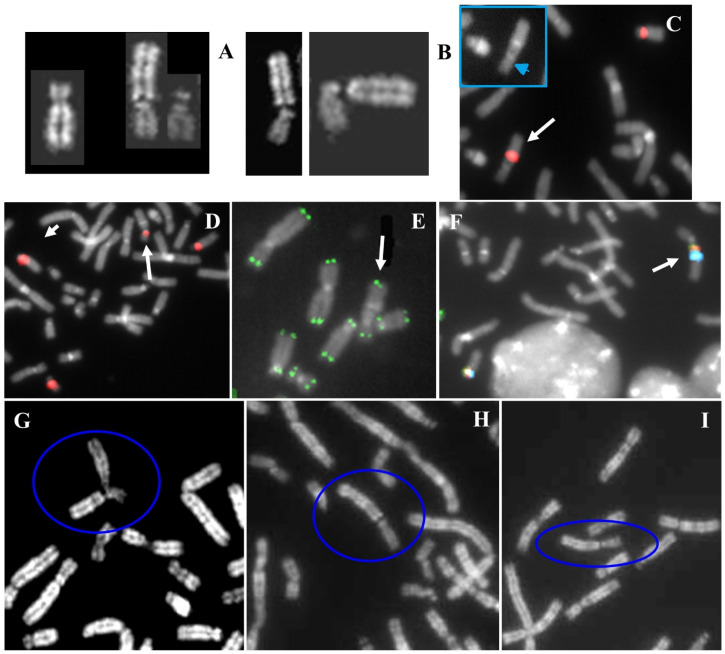
Telomeric case. (**A**) QFQ banded chromosomes of the proband. From left to right: normal 18, derivative t(22;18), normal 22. (**B**) Proband’s partial karyotype showing an apparent breakage of stalk 22. (**C**) FISH with D18Z1 probe: the arrow indicates the translocation. The detail shows the same image without the probe, which shows the absence of primary constriction at the inactive centromere of 18 and the presence of the unique primary constriction of chromosome 22 (arrowed head). (**D**) FISH with D14Z1/D22Z1 probe: the normal chromosomes 14 and 22 and the harrowed derivative 22;18. (**E**) FISH with common telomeric sequences shows the presence of signal at 22q and 18q and the absence of interstitial signal at reunion point 22p;18p. (**F**) FISH with D18Z1 probe (green signals) and specific 18p subtelomeric probe (red signals). The arrow indicates the translocation. (**G**–**I**) Partial metaphases of proband’s father, blue circles evidence the derivative.

The cytogenetic analysis revealed a translocation between chromosomes 18 and 22: psu dic(22;18)(p13;p11.32) (Figure 6A). The same translocation was identified in the father, while both mother and wife had a normal karyotype The pseudodicentric nature of the derivative has been attested by conventional cytogenetics (unique primary constriction of chromosome 22) (Figure 6A). Then, FISH analysis with D18Z1 probe confirmed the inactivation of chromosome 18′s centromere (Figure 6C). D14Z1/D22Z1 probe hybridize the normal chromosomes 14 and 22 and the derivative t(22;18) (Figure 6D). The breakpoint on the derivative was negative to the common pantelomeric probe (Figure 6E), but positive to the 18p specific subtelomeric probe (Figure 6F). Instability of the NOR region of chromosome derivative was observed both in the proband and his father (Figure 6B,G–I) and also in association with other acrocentric chromosomes (Figure 6G–I). Therefore, the derivative lost the repetitive telomeric sequences of 18p, but preserved the whole 18p arm. The karyotype of the proband was:

45,XY,psu dic(22;18)(p13;p11.32)pat.ish psu dic(22;18)(D18Z1+,D18S552+, all human telomere-; all human telomere-,D14Z1/D22Z1+).

### 2.7. Chromosome 18 “Loves” Acrocentrics? What Is the Supporting Evidence?

**Figure 7:**
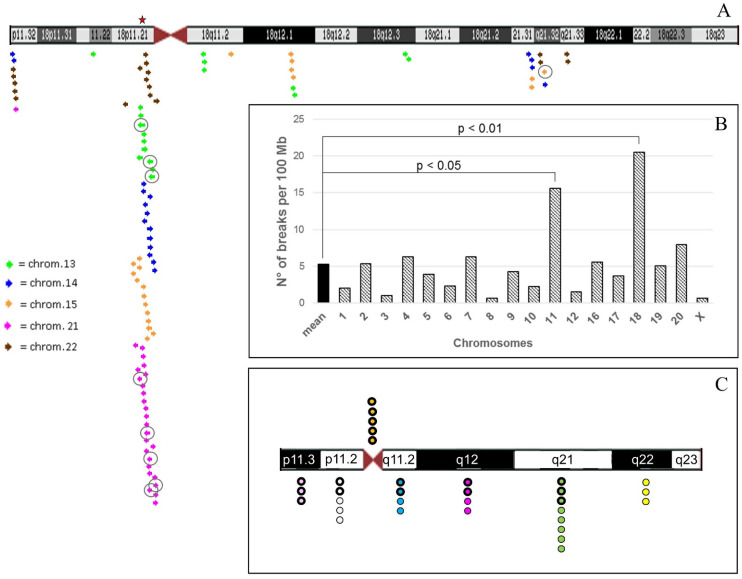
(**A**) Chromosome 18 ideogram modified from UCSC Human Genome Browser—hg19 assembly. The regions of chromosome 18 subjected to analysis are those involved in translocations with acrocentrics. The coloured arrows indicate segmental duplications (duplications of >1000 Bases of Non-RepeatMasked Sequence) with homology 90–98% with acrocentrics, as represented by the inset; the tip of the arrow pointing right indicates the strand +; the tip of the arrow pointing left indicates strand −. The arrows with the circle represent higher homology (98–99%). In particular, 18p11.32 band shows homology with segmental duplications in: 14q11.2, 22q11.1, 22q12.1, 22q13.33 and 21q22.3; 18p11.22 with 13q21.33; 18p11.21 with: 21p11.1-p11.2, 21q11.2, 22q11.1-q11.2, 14q11.1-q11.2, 15q11.2, 13q11.1, 22q13.3, 15q24.3, 14q12, 13q12, and 21q22.3. 18q11.2 with segmental duplications in: 13q14.3 and 15q23; 18q12.1 with 15q22.31, 15q14, 15q26.3, 13q12.11 and 13q14.3; 18q12.3 with 13q12.11; 18q21.31 with 14q23.3, 14q32.2, 14q26 and 15q22.2; 18q21.32 with 22q11.1, 15q23 and 14q31.3; 18q21.33 with 22q12.3. The red star indicates a ~47 Kb sequence with 93% identity between 18p11.21 (BAC RP11-681B3) and 21p11.2 satellite DNA I, II, III (BAC CR381670 [3]). (**B**) Histogram reporting the mean breakpoints number/100 Mb (see Tables S2 and S3). (**C**) 300-band resolution ideogram of chromosome 18 and distribution of breakpoints of translocations listed in Table 1 and Table S1. The colors indicate the different cytogenetic bands of chromosome 18 as in Table S1. The thicker edges of the circles show translocations with acrocentrics; the thinnest edges point out translocations with non-acrocentric chromosomes.

A summary of cases collected from three different laboratories is shown in Table 1 and Figure 7C (see details on Tables S1–S3).

We collected 31 cases of rearrangements of chromosome 18, of which 16 involved an acrocentric chromosome (51.6%). Is there any evidence to support the hypothesis that chromosome 18 preferentially recombines with acrocentrics?

Firstly, if by absurdity we assume that all chromosomes have the same chance of rearranging each other, without considering their size, the expected probability that one chromosome 18 will randomly rearrange with another chromosome is 2.22% (1/45, because we consider diploid genome). Therefore, the probability that one chromosome 18 will randomly rearrange with one acrocentric (10 acrocentric chromosomes) is 22.2%, while the probability that one chromosome 18 will randomly rearrange with one non-acrocentric (35 non-acrocentric chromosomes) is 77.7%. Assuming that the 31 observed rearrangements occurred randomly, we can calculate how many are the expected rearrangements for the two categories through a simple proportion: 7 in the case of acrocentrics (31:100 = X:22.2), and 24 for non-acrocentrics (31:100 = X:77.7). The chi-square statistic between observed (16 and 15) and expected (7 and 24) gives a *p*-value 0.017 (significant at *p* < 0.05); therefore, the hypothesis H0 is to be rejected and the rearrangement is not due to chance.

Secondly, considering the number of breaks interesting acrocentrics in relation to the size in Mb of non-acrocentric chromosomes, again chromosome 18 shows the highest number of breaks, which even exceeds that of the well-known recurrent translocation t(11;22) (20.51 vs 15.56 breaks per 100 Mb, respectively) (Tables S2 and S3, Figure 7B and Figure S2). Statistical analysis between number of breaks per 100 Mb of each chromosome and the mean value of breaks per 100 Mb of all chromosomes (mean = 5.3) revealed that only chromosomes 18 and 11 showed a statistically significant difference, more evidenced for chromosome 18 (Figure 7B, chi-square test). It should also be noted that chromosome 18 has the shortest p arm among all non-acrocentrics (Table S2).

On the other hand, we searched for homologous sequences between 18 and the acrocentrics that could explain this high recombination frequency, which can trigger a Non Allelic Homologous Recombination (NAHR) mechanism. We searched for segmental duplications at the breakpoint regions of chromosome 18 interested in translocations with acrocentrics, as reported in Figure 7C and Table S1. The highest number of segmental duplications is in 18p11.21: we found a total of 68 segmental duplications distributed among all acrocentric chromosomes, with at least 90–98% of homology, of which eight with 98–99%. Chromosome 21, as well as being the most enriched in duplications, showed a sequence of 47 Kb at 21p11.2 satellite DNA I, II, III, with 93% identity to 18p11.21 (Figure 7A). However, since we also found numerous segmental duplications homologous to non-acrocentric chromosomes in these breakpoints, probably the explanation of this phenomenon may involve but not to be limited to the NAHR mechanism.

Then, we performed a qualitative 2D-FISH analysis with D18Z1, D14Z1/D22Z1, and D13Z/D21Z probes using DXZ1 and DYZ3 as control probes, in order to evaluate whether chromosome 18 is preferentially located closer to one acrocentric than to a control chromosome. We observed the behaviour of centromere 18 on interphase nuclei of peripheral blood lymphocytes of six phenotypically normal subjects (three females and three males), collecting two-dimensional data in terms of overlapped/close or distant signals between centromere 18 (D18Z1) and signals of acrocentric probes or control probes (Figure S3 and Table S4).

Basing on these observations, chromosome 18 seems to be more frequently close to acrocentric chromosomes; in fact, there are apparently more cells in which the centromere of 18 locate close to the centromere of the acrocentrics than those in which the two signals are distant and vice versa. Moreover, there are much more cells in which signals for the centromere of 18 are distant from a control probe for a non-acrocentric chromosome respect to the number of cells in which these signals locate close. However, since there is an intrinsic bias in the analysis, due to the unfair comparison of the signals (the acrocentric probes recognize 4 centromeres at a time instead of two), we cannot perform a statistical analysis of these data.

Recently, Hi-C analysis (high-throughput chromosome conformation capture) has revealed spatial segregation of chromosomes in the human genome into distinct sub-compartments [10]. Hi-C identified two nuclear compartments, defined as A and B, correlated with actively transcribed open chromatin and more silent compact chromatin, respectively, and very closely correlated with early- and late-replicating DNA. Here, we used the Juicebox tool [11] in order to explore published Hi-C data generated from the human lymphoblastoid (GM12878) cell line, specifically on acrocentric chromosomes and chromosome 18 (Figure S4A–E). We observed that more colored areas were associated to loci within the same subcompartment of the pericentromeric region of chromosome 18 and acrocentric chromosomes, especially B1 and B2, while with non-acrocentric chromosomes this preference is not so obvious (some examples in Figure S5). This observation would prove once again the presence of greater contact between these regions.

## 3. Discussion

During conventional genetic screening done in three independent laboratories, we noticed an intriguing enrichment of reciprocal translocations between the centromere of chromosome 18 and the centromeric or pericentromeric regions of the acrocentric chromosomes. In addition, we describe a case of telomere fusion between chromosome 18 and the acrocentric 22.

Literature reports that 16% of all translocations occur between the centromere of chromosome 18 and the p arm/cen of acrocentrics [6]. However, few articles describe this type of rearrangement; the first dates back to 1988 [12]. Cooper et al. [13] reported a woman with a balanced translocation t(13;18)(13q;18q)(13p;18p) identified after three abortions, showing chromosomal abnormalities due to meiotic segregation errors. The description is similar to our case number 4. Wang et al. [14] identified two cases with t(18;21), similar to our case number 5. McGheen et al. [15] described one case in prenatal diagnosis with 18p- deletion due to a translocation t(18;22)(q10;q10) similar to our case number 2. Sebold et al. [16] reported 91 patients with del(18p), eleven of which had 18p- as the result of an unbalanced translocation involving an acrocentric chromosome. The same authors reviewed 106 patients 18p- with the aim to provide genotype-phenotype correlations, stating that approximately half of the cases had breakpoints within the centromeric region [7]. The remaining breakpoints are scattered along the rest of the p arm of the chromosome [9]. A summary of literature is reported in Table S5.

Taken globally, these observations suggested that the centromere of chromosome 18 could interact preferentially with the centromeres of acrocentric chromosomes. As a matter of fact, this behavior does not seem to occur by chance, as we found a high density of sequences with high homology, especially in 18p11.21, which can likely trigger NAHR in these centromeric and pericentromeric regions. However, the presence of segmental duplications homologous to non-acrocentric chromosomes makes us speculate on other mechanisms contributing to produce this significant preference.

Native human centromeres are formed by regions of alpha satellite, a repetitive DNA that is defined by a 171 bp monomeric sequence unit. The number and order of monomers confers chromosome specificity, with the exceptions of two pairs of acrocentrics: 13/21, which share the same alpha satellite D13Z1/D21Z1, and 14/22 sharing the D14Z1/D22Z1 [17,18]. Although the function of centromere and its structure have been known for a long time, there are still many aspects that are not clear, defining the so-called dark side of centromeres [1]. Indeed, centromere analysis is still largely limited by the lack of appropriate DNA sequencing technologies for such large repetitive sequences, making centromere sequences the dark matter of the human genome. Because of these technical limitations, it is still unclear if the chromosomal breakpoints observed at centromeric regions are actually at the centromeres per se or at the surrounding pericentromeric, heterochromatin-rich regions.

Therefore, the presence of thousands of repetitive sequences makes the centromere a fragile region subject to breakage [18]. Indeed, their replication might represent a demanding job that generally makes this region unstable, prone to replication errors and to recombination due to the formation of secondary structures [19]. The high DNA sequence similarity between centromere repeats drive recombination events between homologous regions located on different chromosomes by NAHR during DNA repair of the break. Rearrangements at the centromeric region are well described in both genetic diseases and cancer [1,9]. In particular, breaks in chromosome 18 centromere have been described in breast cancer, colorectal and pancreatic carcinomas, and melanoma [20]. However, the high incidence of chromosome 18 alterations involving centromeric regions cannot be explained only by its organization consisting of large alpha satellite arrays, because this feature is shared by centromeres of all human chromosomes.

In addition, the preferential recombination between centromere 18 and acrocentric’ centromeres feeds the suspicion that chromosome 18 has much more in common with acrocentrics. One explanation could be that they share the same alphoid DNA belonging to Suprachromosomal Family 2 (SF2), together with chromosomes 2, 4, 8, 9 and 20 [17]. However, why do translocations between chromosomes 2, 4, 8, 9, and 20 appear less frequent than Robertsonian translocations or chromosome 18 and acrocentric translocations? A possible speculation might be that these rearrangements can result in balanced and unbalanced translocations and the unbalanced translocations of chromosome 18 with acrocentric generally lead to the loss of 18p that has a higher fitness than the loss of 2p, 4p, 8p, 9p or 20p. A critical role for these alphoid sequences was supported by evolutionary considerations. Indeed, five paralogous regions are present in 21q22.1, 21q11.1, 2q21, 18p11 and 13q11 that seem to result from duplication in great apes [21]. The authors proposed an inter-chromosomal duplication occurred in two steps in great apes after the divergence of orangutan. Firstly, the 21q22.1 copy was transposed towards the 2q21 in primates. The second step consists of transposition to other pericentromeric regions of HSA18, HSA13, and HSA21 [21].

On the other hand, the recent development of chromosome conformation capture (3C) techniques, including Hi-C, enabled the study of 3D genome architecture, providing a way to divide chromosomes into domains by measuring contact probabilities between chromosomal segments [22]. There are at least five “subcompartments” defined by their long-range interaction patterns, both within and between chromosomes: both A1 and A2, highly enriched for open early replicating chromatin, are gene dense, have highly expressed genes, and are depleted at the nuclear lamina (NL) and at nucleolus-associated domains (NADs). The other three interaction patterns (labeled B1, B2, and B3) are enriched for closed late replicating chromatin. In particular, nearly half of the B1 subcompartment, which represents facultative heterochromatin, coincides with NADs, while B2 includes 62% of pericentromeric heterochromatin and is enriched at the NL and at NADs [23].

Interestingly, interrogating Hi-C data generated from the human lymphoblastoid (GM12878) cell line, we found that about 70% of the whole chromosome 18 belongs to B2 subcompartment, while the p-arm is about at 45% B1, 45% B2 and only 10% A2, therefore reinforcing the idea that the p-arm of chromosome 18 resides in compartments associated with the nucleolus, as also occurs for the rDNA regions of acrocentrics. These observations are in agreement with our data obtained by 2D-FISH.

However, others have noted a peculiar behavior of chromosome 18. Indeed, the previous data of chromosome painting that found chromosome 18 preferentially located at the nuclear periphery and chromosome 19 in the nuclear interior [24], matches with the very high density of large lamina-associated domains (LADs) of the former, in contrast to the latter, which has only a few LADs [25].

In conclusion, clinical cases statistics obtained from three independent Medical Genetics Labs demonstrate that chromosome 18 preferentially recombines with acrocentrics. In this paper, we tried to explain the involved mechanisms, but certainly do not yet have an answer to this curious phenomenon, that until now no one to our knowledge had ever noticed. Despite the 3D organization of mammalian chromatin having been described more than 30 years ago, we just begin to uncover the molecular characteristics of the NADs and to understand the role of the nucleolus in genome organization and function: we suspect that chromosome 18 may play a role in this mechanism.

## 4. Materials and Methods

### 4.1. Chromosome Analysis

The cases were collected from three Medical Genetics Labs and applied the standard methods to conduct the chromosome analysis, following the Italian Society of Human Genetics (SIGU) guidelines [26] and subsequent modifications online, https://www.sigu.net/show/documenti/5/1/linee%20guida%20e%20raccomandazioni?page = 1 (25 February 2014).

Briefly, peripheral blood metaphases were obtained from phytohaemagglutinin-stimulated lymphocytes. Amniotic fluid was cultured using standard techniques and the chromosomal preparations were performed both in suspension and in situ from independent cultures. Chromosome analysis was carried out applying QFQ or GTG banding according to routine procedures, and the karyotype was expressed following the guidelines of the International System for Cytogenomic Nomenclature 2020 (ISCN 2020) [27]. All images were captured at 100× magnification. Some of them were then enlarged photographically.

### 4.2. D-FISH Analysis

Fluorescence in situ hybridization (FISH) was carried out according to the manufacturer’s protocol for the commercial probes and as previously reported for the homemade ones [28].

The following commercial probes were applied: centromeric probes (D13Z1D21Z1, D14Z1 D22Z1, D18Z1; Oncor, Gaithersburg, MD, USA), 18p11.3 probe (D18S552; Vysis, Abbott Park, IL, USA), TUPLE1 probe (22q11.2; Cytocell, Cambridge, UK), WCP 14 probe (Cytocell, Cambridge, UK) and mix for chr11 telomeric sequences (ToTelVysion probes, Vysis, Abbott Park, IL, USA). BAC (Bacterial Artificial Chromosomes) probes were applied: RP11-19M12 (chr18: 14540099-14719440, hg18) and RP11-48H7 (chr18: 15238370-15399385, hg18) (Wellcome Trust Sanger Institute). For interphase FISH: D1321Z1D21Z1, D14Z1D22Z1 (Cytocell, Cambridge, UK), D18Z1, DXZ1 and DYZ3 (Vysis, Abbott Park, IL, USA). All digital images were captured using a Leitz microscope (LeicaDMRA2 or LeicaDM5000B, Leica Microsystems GmbH, Leica Microsystems, Milan, Italy) equipped with a charge coupled device (CCD) camera (Leica Microsystems) and analyzed by means of various software (Leica CW4000 or Chromowin). All images were captured at 100× magnification. Some of them were then enlarged photographically.

### 4.3. Array Comparative Genomic Hybridization (Array-CGH)

Array Comparative Genomic Hybridization analysis was performed using SurePrint G3 Human CGH plus SNP Microarray following the manufacturer’s instructions (Agilent Technologies, Palo Alto, CA, USA). Genomic DNA was extracted using Wizard Genomic DNA Purification Kit (Promega TM, Mannheim, Germany) or GenElute Blood Genomic DNA kit (Sigma, Darmstadt, Germany), according to the manufacturer’s instructions. DNA concentration was determined on a NanoDrop ND-1000 spectrophotometer (NanoDrop Technologies, Berlin, Germany). DNA control reference was provided by Agilent. The array was scanned at 3-µm resolution using Agilent microarray scanner and analyzed using Agilent microarray scanner and analyzed using Feature Extraction v10.7 in order to read scanner image and Genomic Workbench v7.0 (Agilent Technologies, Palo Alto, CA, USA) to analyze copy number variations. Significant chromosomal aberration was determined using the algorithm ADM-2 (threshold, 5.0; absolute minimum average log2 ratio, 0.20; with at least three or more consecutive probe sets; see more detailed in [29]).

### 4.4. Microsatellite Analysis

The analysis of parental origin was performed on genomic DNA with chromosome 18′s microsatellites: D18S1140 and D18S59 (18p11.32), D18S63 (18p11.31), D18S464 (18p11.22) and D18S53 (18p11.21) (Figure S1). All the microsatellite markers used in this study were selected consulting UCSC Genome Browser.

### 4.5. Methylation-Specific PCR

The CpG WIZ Prader-Willi/Angelman Amplification Kit was used for methylation-specific PCR, following the manufacturer’s instructions (Chemicon, Fisher Scientific, Pittsburgh, PA, USA). Briefly, the first step was the bisulfite modification of the DNA samples, followed by PCR amplification with specific primers designed to distinguish methylated from unmethylated DNA. Finally, a polyacrylamide gel analysis was performed.

### 4.6. UCSC Genome Browser Analysis

Segmental duplications reported in Figure 7A were collected from the UCSC genome browser hg19 assembly. Available online: https://genome.ucsc.edu/cgi-bin/hgGateway (accessed on 30 November 2020) selecting only the regions of chromosome 18 involved in translocations with acrocentrics. Segmental duplications track can be found in the Repeats group.

### 4.7. Bioinformatics Analysis

To explore the interaction between chromosome 18 centromeric region and acrocentric chromosomes, we made use of the Hi-C data for lymphoblastoid GM12878 cell line from the ENCODE archive. GM12878 Hi-C data were then loaded in the standalone version of the Juicebox tool [11]. Available online: https://github.com/aidenlab/Juicebox/wiki (accessed on 27 February 2021) to explore putative chromosome-chromosome interactions.

### 4.8. Statistics

Statistical analyses were carried out performing Yates’ chi-square test on raw data, by means of Excel spreadsheet (Microsoft Corporation, Redmond, WA, USA). *p* value < 0.05 was considered statistically significant.

## Figures and Tables

**Table 1:** Summary of cases described in this work.

	Reciprocal Translocations Involving 18	Chromosome Translocations between 18 and One Acrocentric		Chr. Translocations between 18 and Non-Acrocentric
		Break at the Centromere of 18	Break Not at the Centromere of 18	
Laboratory 1	11	2 (Chr 22) 1 (Chr 15)	1 (Chr 13)	5
			1 (Chr 14)	
			1 (Chr 22)	
Laboratory 2	7	1 (Chr 13)	1 (Chr 13)	4
			1 (Chr 14)	
Laboratory 3	13	1 (Chr 21)	2 (Chr 13)	6
			3 (Chr 14)	
			1 (Chr 22)	
Total	31	5 (16.1%)	11 (35.5%)	15 (48.4%)

## Data Availability

Data available on request due to restrictions eg privacy or ethical.

## Supplementary Materials

The following are available online at https://www.mdpi.com/article/10.3390/ijms22115637/s1.

## References

[1] Barra V., Fachinetti D. (2018). The dark side of centromeres: Types, causes and consequences of structural abnormalities implicating centromeric DNA. Nat. Commun..

[2] Wang J.C., Hajianpour A., Habibian R. (2009). Centromeric alpha-satellite DNA break in reciprocal translocations. Cytogenet. Genome Res..

[3] Jarmuz-Szymczak M., Janiszewska J., Szyfter K., Shaffer L.G. (2014). Narrowing the localization of the region breakpoint in most frequent Robertsonian translocations. Chromosome Res..

[4] Warburton D. (1991). De novo balanced chromosome rearrangements and extra marker chromosomes identified at prenatal diagnosis: Clinical significance and distribution of breakpoints. Am. J. Hum. Genet..

[5] Hamerton J.L., Canning N., Ray M., Smith S. (1975). A cytogenetic survey of 14,069 newborn infants. I. Incidence of chromosome abnormalities. Clin. Genet..

[6] Schinzel A. (2001). Catalogue of Unbalanced Chromosome Aberrations in Humans.

[7] Schaub R.L., Reveles X.T., Baillargeon J., Leach R.J., Cody J.D. (2002). Molecular characterization of 18p deletions: Evidence for a breakpoint cluster. Genet. Med..

[8] Turleau C. (2008). Monosomy 18p. Orphanet J. Rare Dis..

[9] Hasi-Zogaj M., Sebold C., Heard P., Carter E., Soileau B., Hill A., Rupert D., Perry B., Atkinson S., O’Donnell L. (2015). A review of 18p deletions. Am. J. Med. Genet. C Semin. Med. Genet..

[10] Rao S.S., Huntley M.H., Durand N.C., Stamenova E.K., Bochkov I.D., Robinson J.T., Sanborn A.L., Machol I., Omer A.D., Lander E.S. (2014). A 3D map of the human genome at kilobase resolution reveals principles of chromatin looping. Cell.

[11] Durand N.C., Robinson J.T., Shamim M.S., Machol I., Mesirov J.P., Lander E.S., Aiede E.L. (2016). Juicebox Provides a Visualization System for Hi-C Contact Maps with Unlimited Zoom. Cell Syst..

[12] Ginzburg I.A., Lisichenko O.V., Kitaĭnik G.P. (1988). Case of unbalanced translocation (18, 22) in a child with congenital mental retardation. Tsitol. Genet..

[13] Cooper P.J., Towe C., Crolla J.A. (1993). A balanced whole arm reciprocal translocation resulting in three different adverse pregnancy outcomes. J. Med. Genet..

[14] Wang J.C., Nemana L., Kou S.Y., Habibian R., Hajianpour M.J. (1997). Molecular cytogenetic characterization of 18;21 whole arm translocation associated with monosomy 18p. Am. J. Med. Genet..

[15] McGhee E.M., Qu Y., Wohlferd M.M., Goldberg J.D., Norton M.E., Cotter P.D. (2001). Prenatal diagnosis and characterization of an unbalanced whole arm translocation resulting in monosomy for 18p. Clin. Genet..

[16] Sebold C., Soileau B., Heard P., Carter E., O’Donnell L., Halle D.E., Cody J.D. (2015). Whole arm deletions of 18p: Medical and developmental effects. Am. J. Med. Genet. A.

[17] McNulty S.M., Sullivan B.A. (2018). Alpha satellite DNA biology: Finding function in the recesses of the genome. Chromosome Res..

[18] Sullivan L.L., Sullivan B.A. (2020). Genomic and functional variation of human centromeres. Exp. Cell Res..

[19] Carvalho C.M., Lupski J.R. (2016). Mechanisms underlying structural variant formation in genomic disorders. Nat. Rev. Genet..

[20] Alsop A.E., Teschendorff A.E., Edwards P.A. (2006). Distribution of breakpoints on chromosome 18 in breast, colorectal, and pancreatic carcinoma cell lines. Cancer Genet. Cytogen..

[21] Golfier G., Chibon F., Aurias A., Chen X.N., Korenberg J., Rossier J., Potier M.C. (2003). The 200-kb segmental duplication on human chromosome 21 originates from a pericentromeric dissemination involving human chromosomes 2, 18 and 13. Gene.

[22] Kempfer R., Pombo A. (2020). Methods for mapping 3D chromosome architecture. Nat. Rev. Genet..

[23] Dillinger S., Straub T., Németh A. (2017). Nucleolus association of chromosomal domains is largely maintained in cellular senescence despite massive nuclear reorganisation. PLoS ONE.

[24] Cremer M., von Hase J., Volm T., Brero A., Kreth G., Walter J., Fischer C., Solovei I., Cremer C., Cremer T. (2001). Non-random radial higher-order chromatin arrangements in nuclei of diploid human cells. Chromosome Res..

[25] Kind J., Pagie L., de Vries S.S., Nahidiazar L., Dey S.S., Bienko M., Zhan Y., Lajoie B., de Graaf C.A., Amendola M. (2015). Genome-wide maps of nuclear lamina interactions in single human cells. Cell.

[26] Associazione Italiana di Citogenetica Medica (1995). Diagnostica Citogenetica, Consensus. Analysis.

[27] McGowan-Jordan J., Hastings R.J., Moore S. (2020). International Standing Committee on Human Cytogenomic Nomenclature. ISCN: An International System for Human Cytogenomic Nomenclature.

[28] Lissoni S., Baronchelli S., Villa N., Lucchini V., Betri E., Cavalli P., Dalpra L. (2009). Chromosome territories, X; Y translocation and Premature Ovarian Failure: Is there a relationship?. Mol. Cytogen..

[29] Redaelli S., Maitz S., Crosti F., Sala E., Villa N., Spaccini L., Selicorni A., Rigoldi M., Conconi D., Dalpra L. (2019). Refining the Phenotype of Recurrent Rearrangements of Chromosome 16. Int. J. Mol. Sci.

