# Supplementary material for: Human Chromosome 18 and Acrocentrics: A Dangerous Liaison
Source: Int J Mol Sci. 2021 May 26;22(11):5637. doi: 10.3390/ijms22115637 (PMC8198063; doi:10.3390/ijms22115637)
Supplement: Supplementary file 1 [file ijms-22-05637-s001.zip › Supplementary figures_rev.pdf]

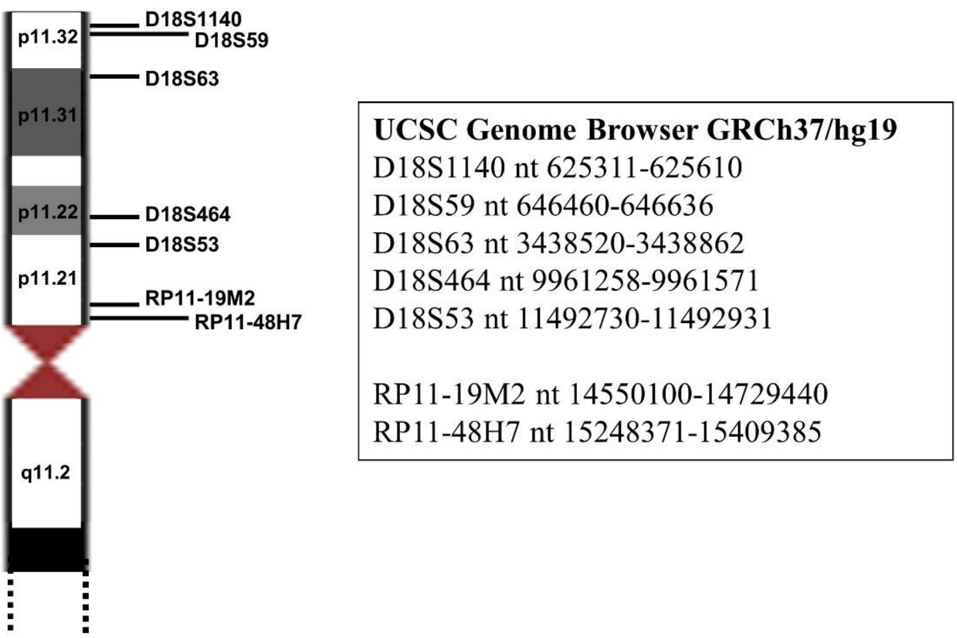

Figure S1. Chromosome 18 ideogram with probes and microsatellites used in this study.

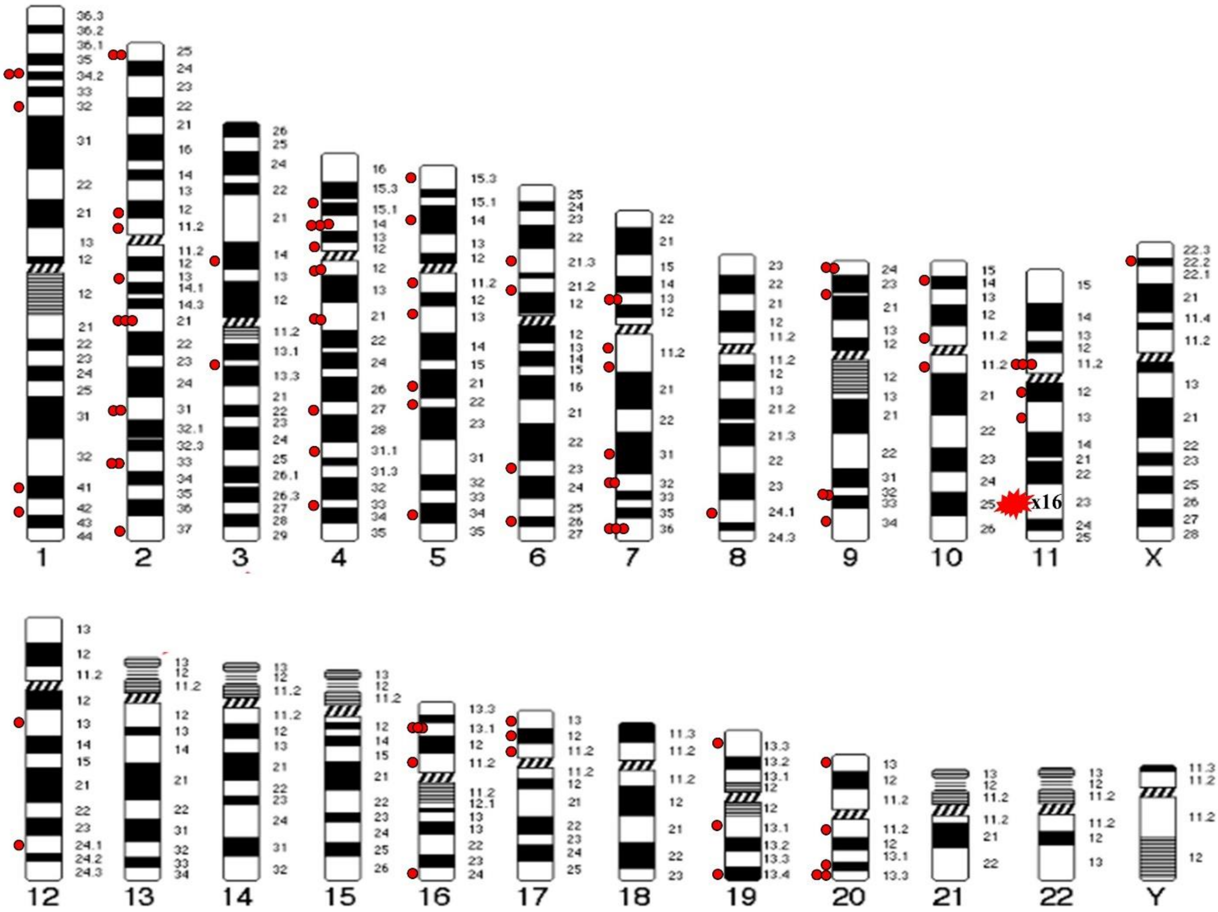

Figure S2. Chromosome ideograms showing the breakpoints of translocations between non-acrocentric chromosomes with one acrocentric chromosome, collected in the three centers in the period from 1999-2020, as reported in Table S3. Data

for chromosome 18 are excluded. Red circles represent a specific translocation; the cloud on chromosome 11 represents the recurrent t(11;22).

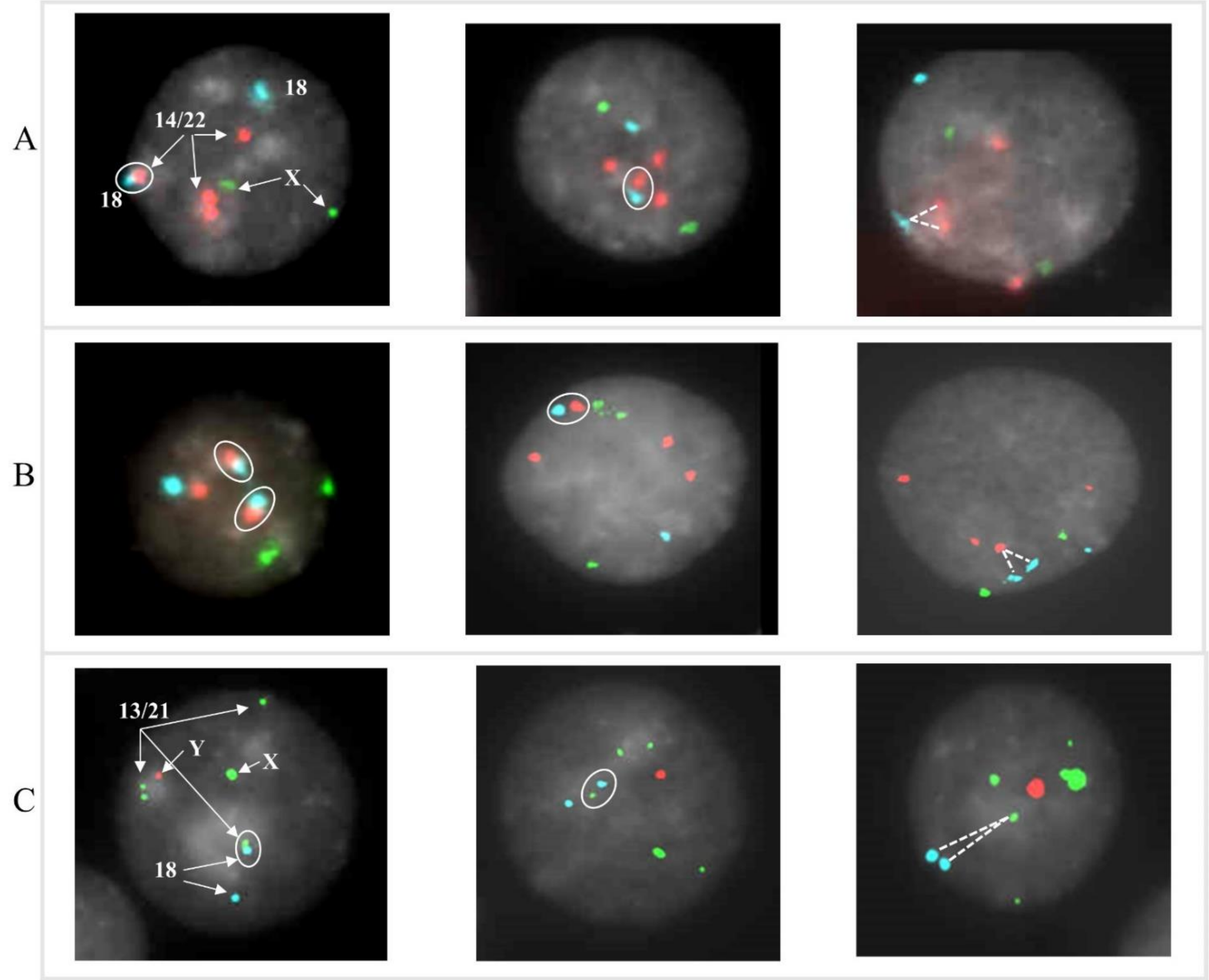

Figure S3. Examples of signal patterns on normal nuclei of females **(A, B)** (D14Z1/D22Z1 and D18Z1) and male **(C)** (D13Z1/D21Z1). The arrows with numbers and letters indicate the corresponding chromosomes. Probes: D13Z1/D21Z1 (green), D14Z1/D22Z1 (red), D18Z1 (Spectrum Aqua), DXZ1 (Spectrum Green), DYZ3 (Spectrum Red). Left and middle panels: examples of overlapped and close signals (white circles); right panel: examples of distant signals (white dashed lines).

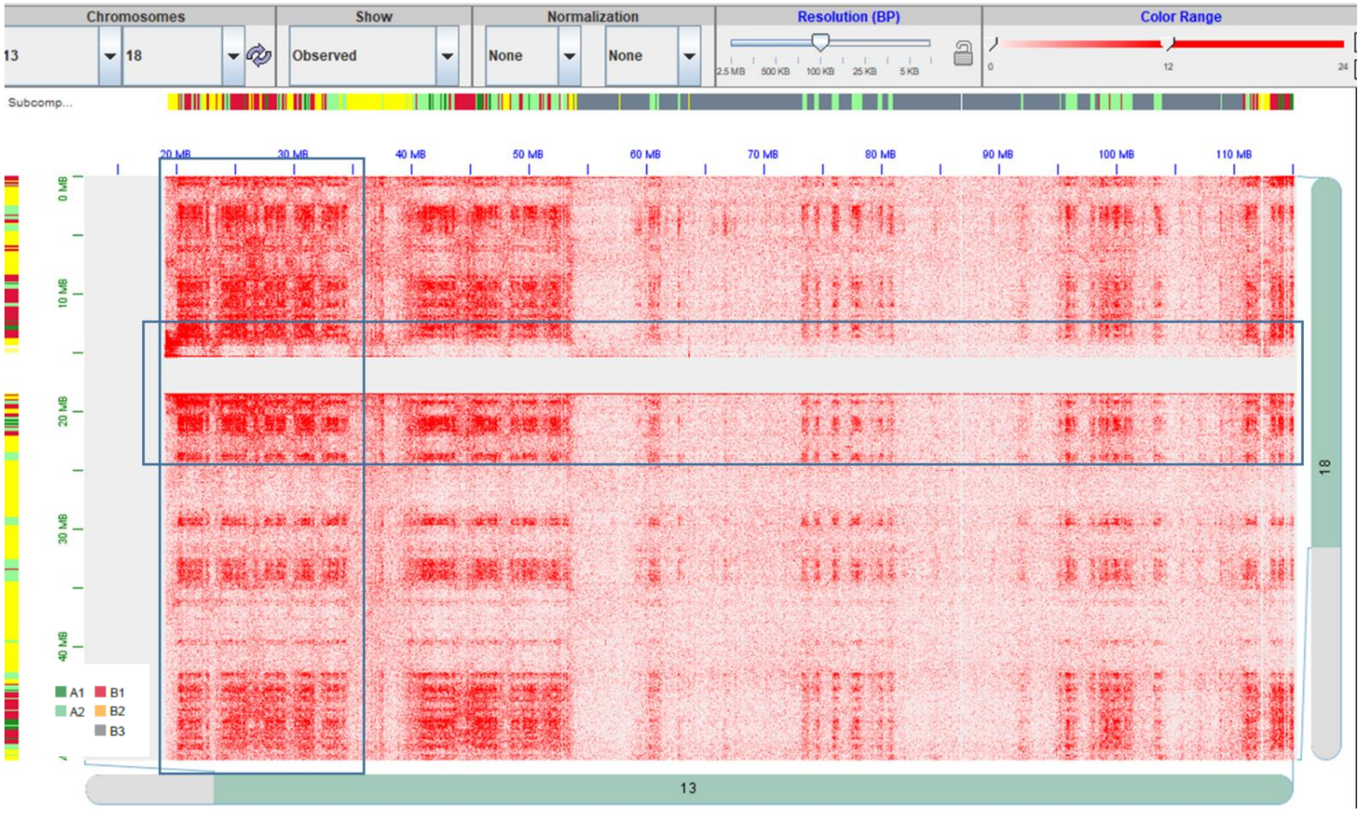

Figure S4.A.

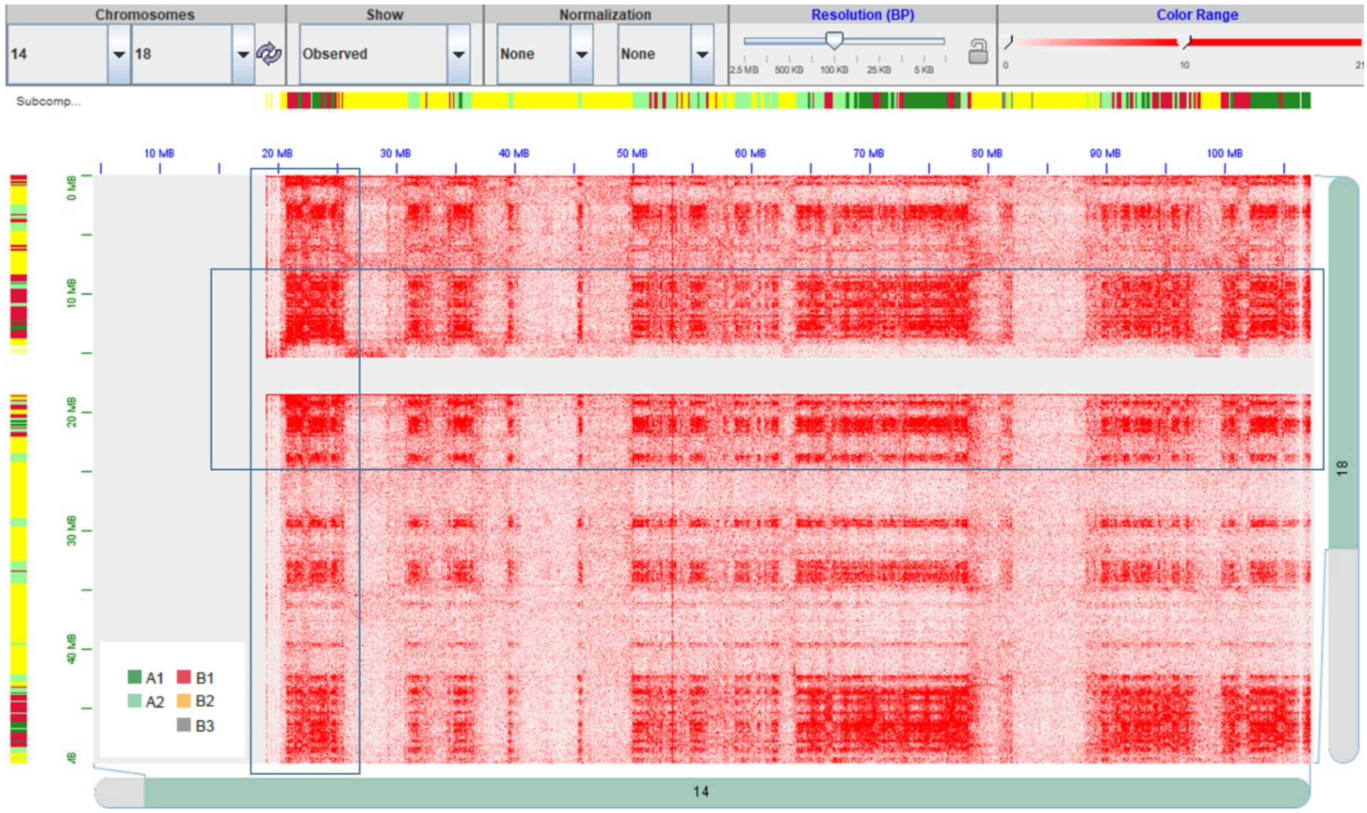

Figure S4.B.

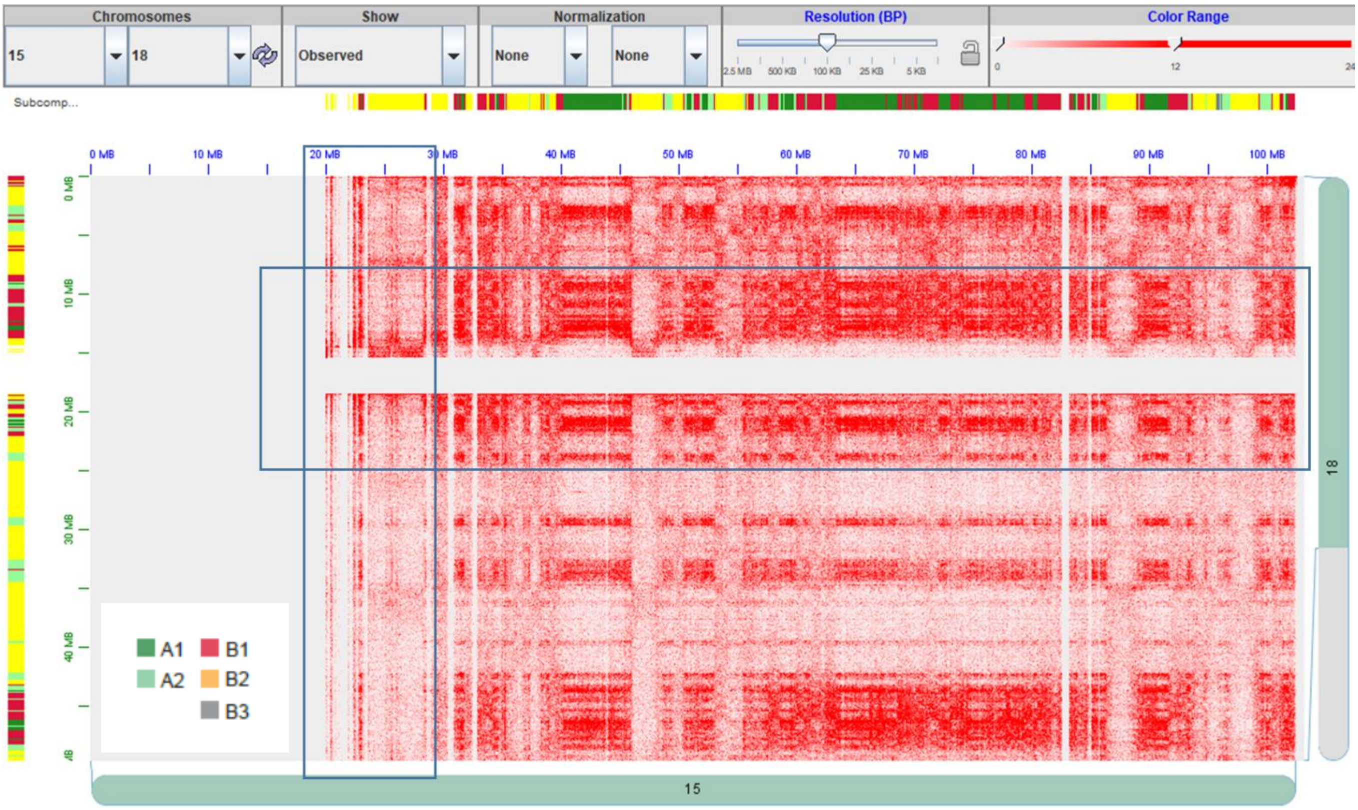

Figure S4.C.

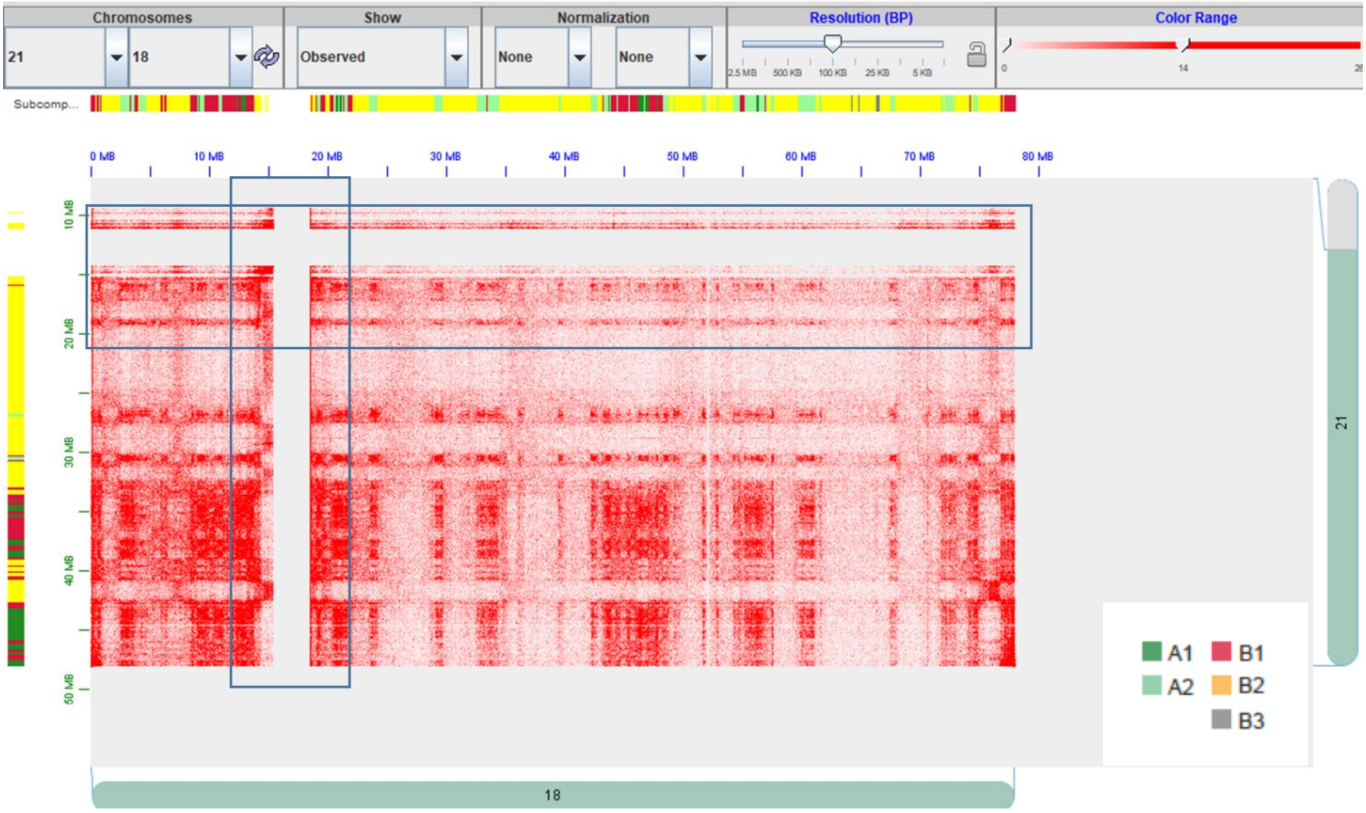

Figure S4.D.

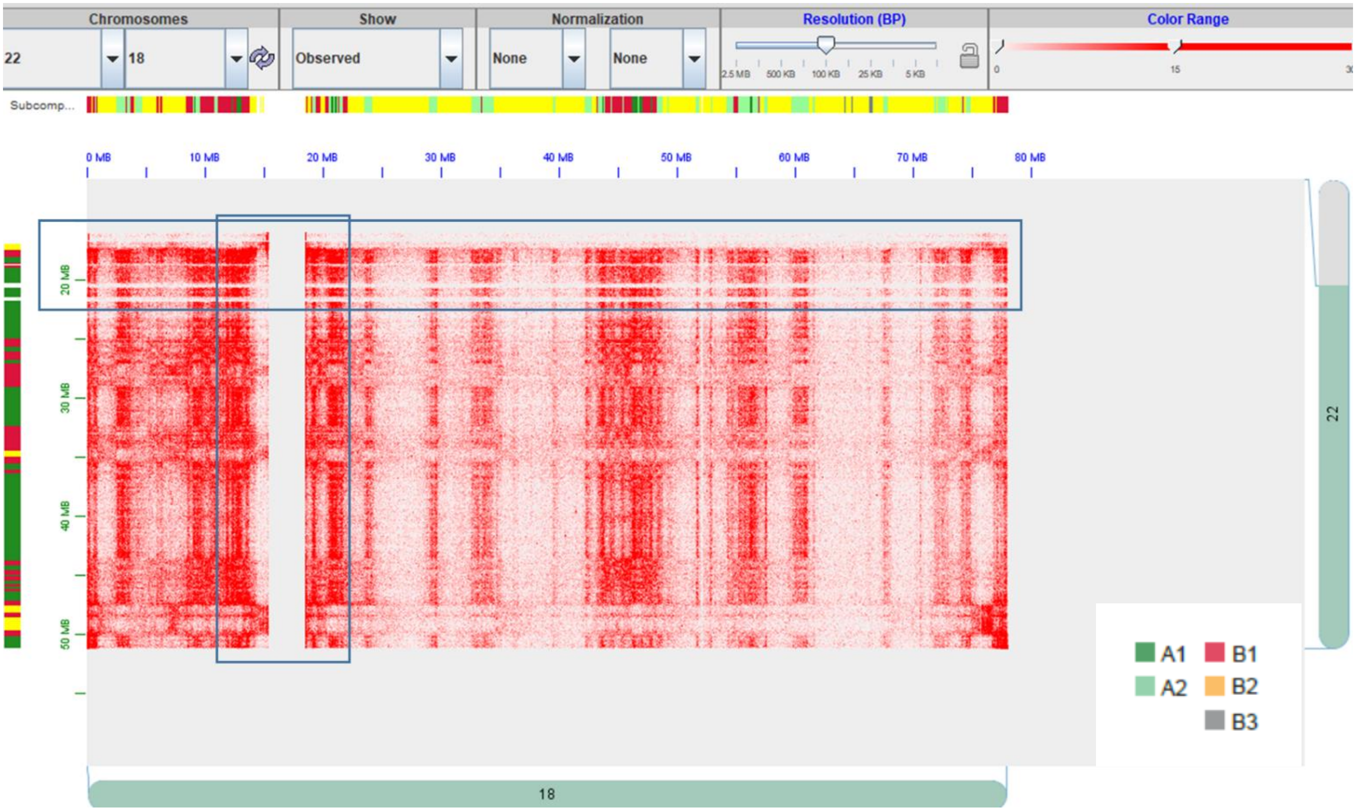

Figure S4.E.

Figure S4. Interchromosomal contact matrices of acrocentrics and chromosome 18. Each panel shows the interactions of one acrocentric chromosome with chromosome 18. Loci within the same subcompartment tend to have the same interchromosomal contact pattern (more colored areas). The centromeric and pericentromeric regions are in rectangles. Resolution: 100 Kb. Color range: 10-15 pixels. The rectangle shows the colors of the sub-compartments A1, A2, B1, B2, B3.

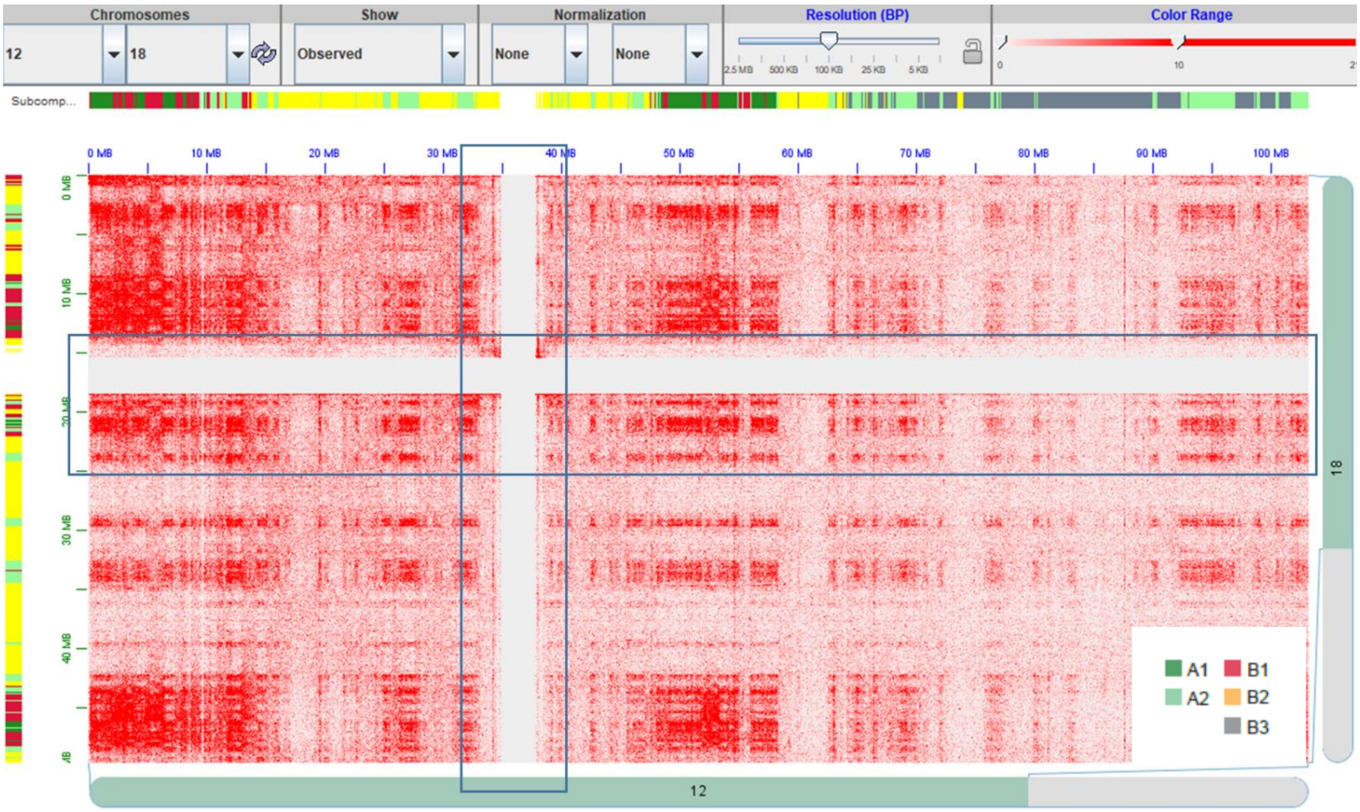

Figure S5.A.

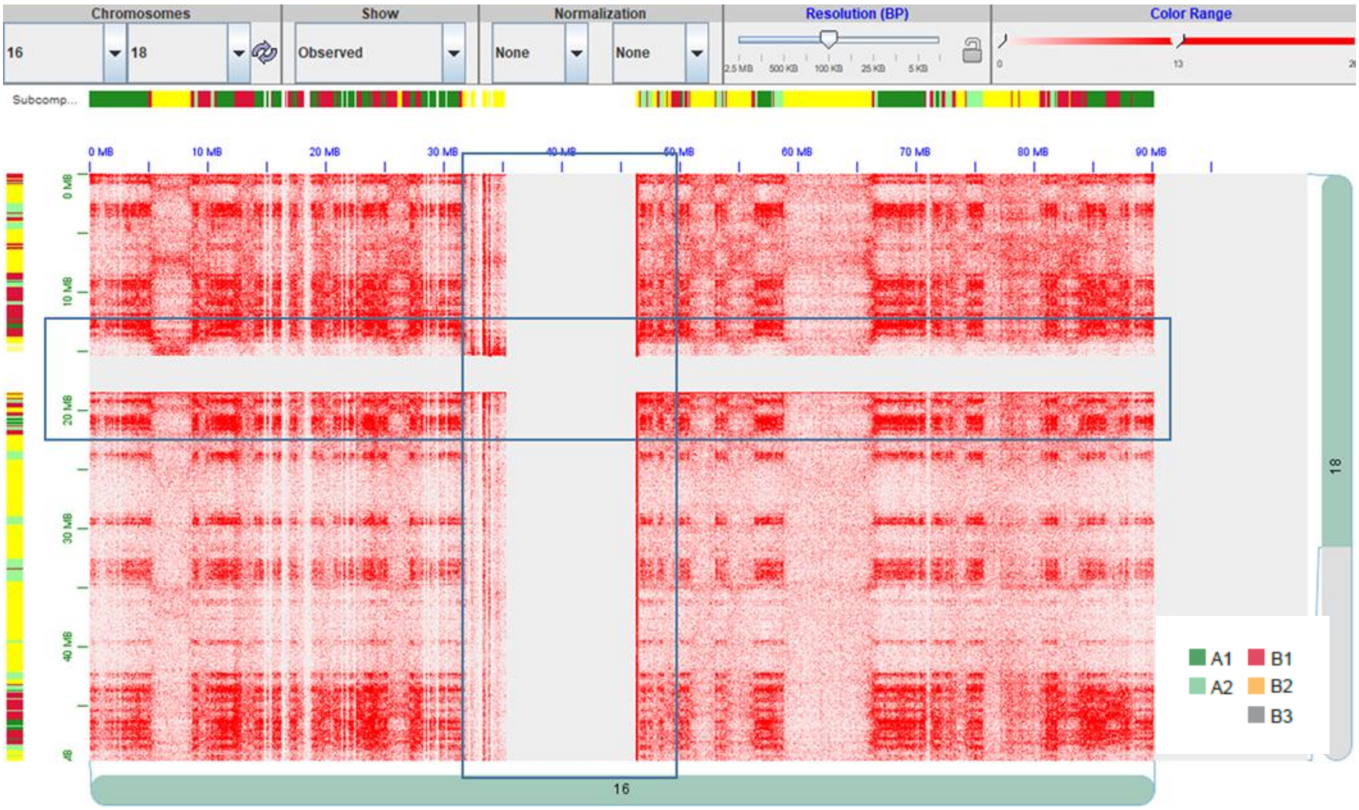

Figure S5.B.

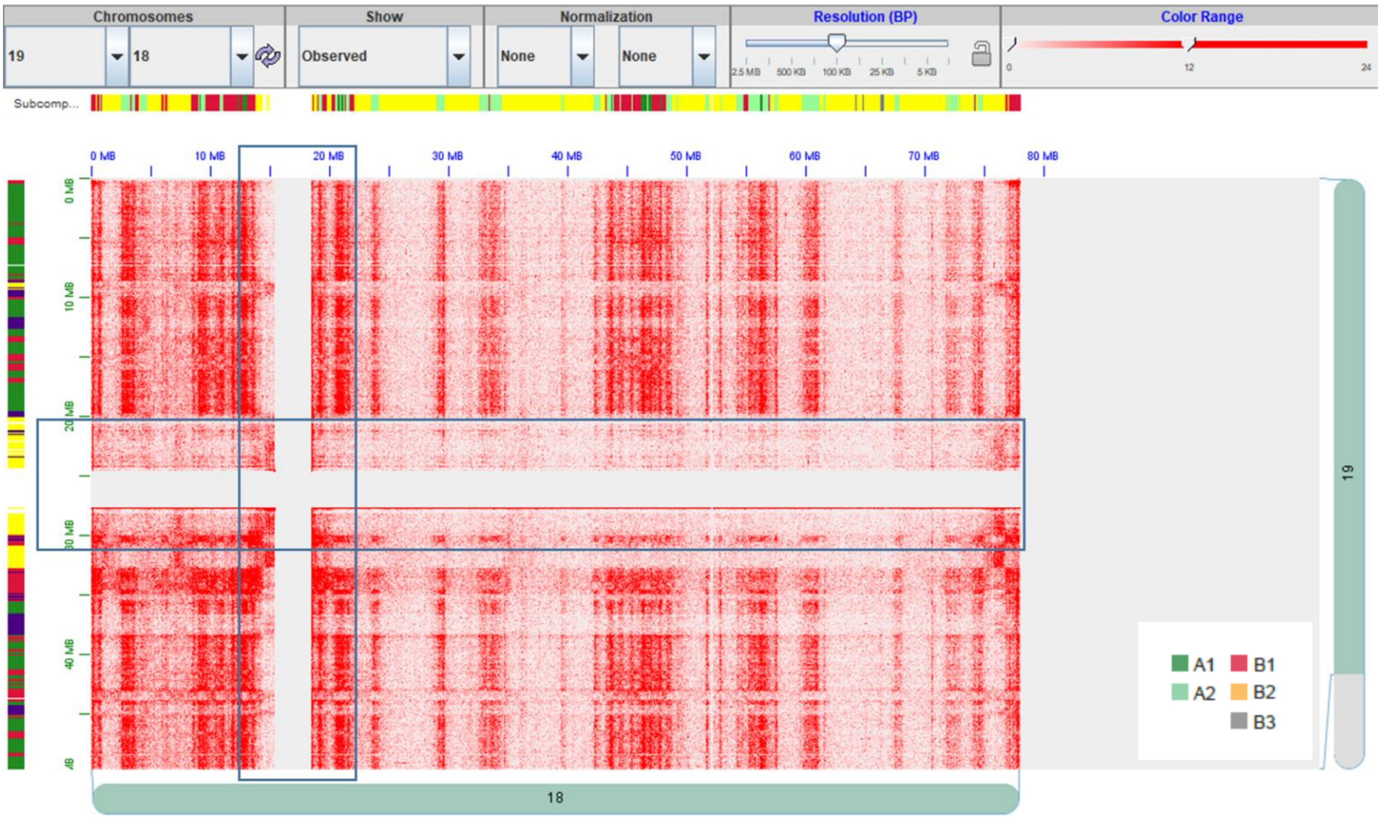

Figure S5.C.

Figure S5. Three examples of interchromosomal contact matrices of non-acrocentrics and chromosome 18. Each panel shows the interactions of one *non*-acrocentric chromosome with chromosome 18. Loci within the same subcompartment tend to have the same interchromosomal contact pattern (more colored areas). The centromeric and pericentromeric regions are in rectangles. Resolution: 100 Kb. Color range: 10-13 pixels. The rectangle shows the colors of the sub-compartments A1, A2, B1, B2, B3.
