# Supplementary material for: Human Chromosome 18 and Acrocentrics: A Dangerous Liaison
Source: Int J Mol Sci. 2021 May 26;22(11):5637. doi: 10.3390/ijms22115637 (PMC8198063; doi:10.3390/ijms22115637)
Supplement: Supplementary file 1 [file ijms-22-05637-s001.zip › Table S1.Rev.pdf]

Table S1. Breakpoint details of translocations of chromosome 18 identified in the 3 centers during the period 1999-2020. The colors indicate the different cytogenetic bands of chromosome 18 as in Fig 7. The brackets in the first column show the case reports described in the results.

	karyotype	breakpoint 18	breakpoint partner
**Lab 1**			
1	46,t(9;18)	q11.2	p13
2	46,t(5;18)	p11.2	q13.3
3	46,t(12;18)	q12.1	q15
4	46,t(4;18)	q21.3	q27
5	46,t(4;18)	q21.3	q25
6 (case 3)	46,der(18)t(18;22)	**q10**	q10
7 (case 2)	45, der(18)t(18;22)	**q10 (?)**	q10 (?)
8* (case 6)	45,psu dic(22;18)	**p11.32**	p13
9	46,t(14;18)	**q21.3**	q24.3
10	46,t(13;18)	**p11.2**	q32
11 (case 4)	46,t(15;18)	**p10**	q11.2
**Lab 2**			
1	46,t(14;18)	**q12**	q21
2	46,t(5;18)	q22	q32
3	46,der(18)(t(4;18)	q22.3	p11.2
4	46,t(18;20)	p11.2	p11.2
5	46,t(4;18)	q21.3	q25
6	46,t(13;18)	**q21.3**	q22.3
7 (case 1)	45,t(13;18)	**q10**	q10
**Lab 3**			
1	46,t(2;18)	q11.2	q34
2	46,t(14;18)	**q21.3**	q21
3* (case 6)	45,psu dic(22;18)	**p11.32**	p13
4	46,t(12;18)	q22	q15
5	46,t(12;18)	q21	q21
6	46,t(8;14;18)(q21.1;q24.3;q11.2)	**q11.2**	8q21.1; 14q24.3
7	46,t(5;18)	p11.22	p15.31
8	46,t(13;18)	**p11.32**	q22
9	mos 46,t(5;18)[40]/46[60]	q12.2	p13.2
10**	46,t(6;13;18;9)	q11.2; **p11.2**	6p12; 13q12.1; 9q13
11	46,t(14;18)	**q12.2**	q13
12	46,t(2;18)	q21.1	q31.1
13 (case 5)	46,+18,der(18;21)	**q10**	q10

*: proband and father
**Patient 10 of Lab 3 has two breaks on chromosome 18
In bold the breakpoints on chromosome 18 involving acrocentrics
