# Supplementary material for: Human Chromosome 18 and Acrocentrics: A Dangerous Liaison
Source: Int J Mol Sci. 2021 May 26;22(11):5637. doi: 10.3390/ijms22115637 (PMC8198063; doi:10.3390/ijms22115637)
Supplement: Supplementary file 1 [file ijms-22-05637-s001.zip › Table S2Rev.pdf]

Table S2. Summary of translocations involving non-acrocentric chromosomes with acrocentric chromosomes collected in the 3 centers in the period from 1999-2020. See Table S3 for details.

**Chromosome**	**Arm length p/q***	**Total Mb****	**N° breaks interesting acrocentrics**	**N° of breaks per 100 Mb**
1	125000000<br>124250621	249	5	2,01
2	93300000<br>149899373	243	13	5,35
3	91000000<br>107022430	198	2	1,01
4	50400000<br>140754276	191	12	6,28
5	48400000<br>132515260	181	7	3,87
6	61000000<br>110115067	171	4	2,34
7	59900000<br>99238663	159	10	6,29
8	45600000<br>100764022	146	1	0,68
9	49000000<br>92213431	141	6	4,26
10	40200000<br>95334747	136	3	2,21
**11**	53700000<br>81306516	**135**	**21**	**15,56**
12	35800000<br>98051895	134	2	1,49
16	36600000<br>53754753	90	5	5,56
17	24000000<br>57195210	81	3	3,7
**18**	17200000<br>60877248	**78**	**16**	**20,51**
19	26500000<br>32628983	59	3	5,08
20	27500000<br>35525520	63	5	7,94
X	60600000<br>94670560	155	1	0,65
mean			*6,6*	*5,3*

* from https://www.biostars.org/p/383786/ accessed on 7 Apr 2021
**UCSC Genome Browser GRCh37/hg19
