# Supplementary material for: Human Chromosome 18 and Acrocentrics: A Dangerous Liaison
Source: Int J Mol Sci. 2021 May 26;22(11):5637. doi: 10.3390/ijms22115637 (PMC8198063; doi:10.3390/ijms22115637)
Supplement: Supplementary file 1 [file ijms-22-05637-s001.zip › Table S3.Rev.pdf]

**Table S3**. Breakpoint details of translocations between non-acrocentric chromosomes and acrocentric chromosomes collected in the three centers in the period from 1999-2020.

| Non-acrocentric chromosomes | Acrocentric chromosomes | | | | |
|---|---|---|---|---|---|
| | 13 | 14 | 15 | 21 | 22 |
| 1p34.1 | | q32.3 | | | |
| 1p34.1 | | | | | q13.1 |
| 1p32 | q32 | | | | |
| 1q41 | | q32.1 | | | |
| 1q42 | | | q11.2 | | |
| 2p25 | | q11.2 | | | |
| 2p25 | | | | q11 | |
| 2p12 | | | | q21 | |
| 2p11.2 | | | | q22.1 | |
| 2q13 | | | q11.2 | | |
| 2q21 | | q24 | | | |
| 2q21.1 | | | q15 | | |
| 2q21.1 | | q24.3 | | | |
| 2q31 | | | q22.1 | | |
| 2q31 | | q31 | | | |
| 2q33 | q14 | | | | |
| 2q33.1 | q32.1 | | | | |
| 2q37.3 | | | q24 | | |
| 3p14 | | | q21 | | |
| 3q13.2 | q22 | | | | |
| 4p15.2 | q21.2 | | | | |
| 4p14 | q14.1 | | | | |
| 4p14 | | q22 | | | |
| 4p14 | | | | q22.1 | |
| 4p12 | | | | p12 | |
| 4q12 | | | | q11.2 | |
| 4q12 | | | | | q13 |
| 4q21.1 | | | | q22.3 | |
| 4q21.1 | q21.2 | | | | |
| 4q27 | | q11.2 | | | |
| 4q31.3 | | | q22.1 | | |
| 4q33 | | | q26.3 | | |
| 5p15.3 | | | | q21 | |
| 5p14 | q32 | | | | |
| 5q11.2 | q12 | | | | |
| 5q13.1 | | | | q21 | |
| 5q21.2 | | | q13 | | |
| 5q22 | | | | | q11.2 |
| 5q34 | | | q24 | | |
| 6p21.3 | q12 | | | | |
| 6p21.1 | | q32.1 | | | |
| 6q23.1 | | | | q22.3 | |

| | | | | | |
|---|---|---|---|---|---|
| 6q26 | | | | q22.2 | |
| 7p13 | q32 | | | | |
| 7p13 | | q13 | | | |
| 7q11.2 | q21 | | | | |
| 7q11.23 | | | q26.1 | | |
| 7q31.1 | | | | q22.3 | |
| 7q32 | q32 | | | | |
| 7q32 | | | | | 13.1 |
| 7q36.1 | | q32.3 | | | |
| 7q36.2 | | p11.1 | | | |
| 7q36.3 | | p11.2 | | | |
| 8q24.1 | | | q11.2 | | |
| 9p24 | | | | | q11 |
| 9p24 | | | | | q13.1 |
| 9p22 | | | | q22.3 | |
| 9q32 | | | p11.1 | | |
| 9q32 | q14 | | | | |
| 9q34.1 | | | | | q11.2 |
| 10p14 | q21.1 | | | | |
| 10p11.2 | | | q21 | | |
| 10q11.1 | | q11.1 | | | |
| 11p11.2 | | q11.1 | | | |
| 11p11.2 | | | q26.1 | | |
| 11p11.2 | q12.1 | | | | |
| 11q12 | q14.1 | | | | |
| 11q13 | | q24.1 | | | |
| 11q23 | | | | | q11 |
| 11q23 | | | | | q11 |
| 11q23 | | | | | q11 |
| 11q23 | | | | | q11.2 |
| 11q23 | | | | | q11.2 |
| 11q23 | | | | | q11.2 |
| 11q23 | | | | | q11.2 |
| 11q23 | | | | | q11.2 |
| 11q23 | | | | | q11.2 |
| 11q23.3 | | | | | q11.2 |
| 11q23.3 | | | | | q11.2 |
| 11q23.3 | | | | | q11.2 |
| 11q23.3 | | | | | q11.2 |
| 11q23.3 | | | | | q11.2 |
| 11q23.3 | | | | | q11.2 |
| 11q23.3 | | | | | q11.2 |
| 12q13 | q12 | | | | |
| 12q24.1 | | | q24 | | |
| 16p13.1 | | | | q22.3 | |
| 16p13.1 | | | q26.3 | | |
| 16p13.1 | | | q15 | | |
| 16p11.2 | q22 | | | | |

| | | | | | |
|---|---|---|---|---|---|
| 16q24.3 | q12.11 | | | | |
| 17p13 | | | q24 | | |
| 17p12 | | | p11.2 | | |
| 17p11.2 | | | p12 | | |
| 19p13.3 | | | q24 | | |
| 19q13.1 | | | | | q11.2 |
| 19q13.4 | | | | q22.2 | |
| 20p13 | | | | q22.1 | |
| 20q11.2 | | | | q22.1 | |
| 20q13.2 | | q24.3 | | | |
| 20q13.3 | | | | | p13 |
| 20q13.3 | | | q22.3 | | |
| Xp22 | | q22 | | | |
