# Supplementary material for: Human Chromosome 18 and Acrocentrics: A Dangerous Liaison
Source: Int J Mol Sci. 2021 May 26;22(11):5637. doi: 10.3390/ijms22115637 (PMC8198063; doi:10.3390/ijms22115637)
Supplement: Supplementary file 1 [file ijms-22-05637-s001.zip › Table S4.pdf]

Table S4. 2D-FISH on nuclei of 72h PHA stimulated blood lymphocytes. All karyotypes were normal.

cases	219-M	215-M	114-F	133-F	134-M	220-F	tot	mean
Probes	D14/22Z1 D18Z1	D14/22Z1 D18Z1	D14/22Z1 D18Z1	D14/22Z1 D18Z1	D13/21Z1 D18Z1	D13/21Z1 D18Z1		
Overlapping/close	36	39	24	23	22	33	177	29,5
distant	14	11	26	27	28	17	123	20,5
total n°	50	50	50	50	50	50	300	
Probes	Control probe/ D18Z1	Control probe/ D18Z1	Control probe/ D18Z1	Control probe/ D18Z1	Control probe/ D18Z1	Control probe/ D18Z1	tot	mean
Overlapping/close	11	15	15	17	12	16	86	14,33
distant	39	35	35	33	38	34	214	35,7
total n°	50	50	50	50	50	50	300	

M: normal male; F: normal female
