# Supplementary material for: Human Chromosome 18 and Acrocentrics: A Dangerous Liaison
Source: Int J Mol Sci. 2021 May 26;22(11):5637. doi: 10.3390/ijms22115637 (PMC8198063; doi:10.3390/ijms22115637)
Supplement: Supplementary file 1 [file ijms-22-05637-s001.zip › Table S5.pdf]

Table S5: Review of literature

Authors	N° of cases	Breakpoint 18	Acrocentric chromosome	Breakpoint acrocentric	Phenotype abn / n	Origin pat / mat	Chromosome abnormality
Prontera et al 2007 [1]	1	p11.1	15	p11.2	1 / 0	n.r.	dic(15;18) del(18p)
Witters et al 2002 [2]	1	q11	13	p11	1/0	n.r.	whole arm not rcp tr. del(18p)
*Ginzburg et al 1988 [3]	1	cen	22	cen	1 / 0	(russian paper)	(russian paper)
Cooper et al 1993 [4]	4 (fam)	p11	13	q11	2° / 2°	mat	whole arm rcp tr.
McGhee et al 2001 [5]	1	q10	22	q10	1 / 0	mat	dic(18;22) del(18p)
Wang et al 1997 [6]	2 ♂	p11.1	21	p11.1	2 / 0	n.r.	both dic(18;21) del(18p)
Binkert et al 1990 [7]	4 (fam)	q12	21	q11	1 / 3§	pat	trisomy 18p
Sebold et al 2015 [8]	11	cen	21 (2/11)	n.r.	11/0	1 mat	del(18p)
Blattner et al 1980 [9]	6 (fam)	q12	13	p13	2 / 4	mat	partial trisomy 18
Gu et al 2011 [10]	1	p11	21	q21	0 / 1	n.r.	rcp tr
Chen et al 2003 [11]	2 (fam)	p11.2	21	q22.3	1 / 1	mat	tris18p, rcp tr
Cotton et al 1993 [12]	6 (fam)	q11	13	q12	5^ / 1	mat	^rcp tr

abn: clinical phenotype, n: normal phenotype, rcp tr: reciprocal translocation, n.r.: not reported, *: from english abstract of a russian paper,
°: mother balanced, 2 aborted fetuses unbalanced and 1 fetus triploid with balanced translocation, §: father, grandmother, uncle
^: 5 prenatal diagnoses of trisomy 18q, trisomy 18p, monosomy 18p

References

1. Prontera, P.; Aiello, V.; Toschi, M.; Turci, A.; Gruppioni, R.; et al. Prenatal diagnosis of a de novo satellited chromosome 18 (18ps) associated with 18p deletion. *Genet Couns* **2007,** *18(3)*, 309-15.
2. Witters, I.; Delattin, P.; Van Schoubroeck, D.; Fryns, J.P. Increased nuchal translucency with de novo acrocentric/18p translocation. *Genet Couns* **2002**, *13(2)*, 203-5.
3. Ginzburg, I.A.; Lisichenko, O.V.; Kitaĭnik, G.P. [Case of unbalanced translocation (18, 22) in a child with congenital mental retardation]. *Tsitol Genet* **1988**, *22(3)*, 52-3.
4. Cooper, P.J.; Towe, C.; Crolla, J.A. A balanced whole arm reciprocal translocation resulting in three different adverse pregnancy outcomes. *J Med Genet* **1993**, *30(5)*, 417-8. doi: 10.1136/jmg.30.5.417.
5. McGhee, E.M.; Qu, Y.; Wohlferd, M.M.; Goldberg, J.D.; Norton, M.E.; Cotter, P.D. Prenatal diagnosis and characterization of an unbalanced whole arm translocation resulting in monosomy for 18p. *Clin Genet* **2001,** *59(4)*, 274-8. doi: 10.1034/j.1399-0004.2001.590410.x.
6. Wang, J.C.; Nemana, L.; Kou, S.Y.; Habibian, R.; Hajianpour, M.J. Molecular cytogenetic characterization of 18;21 whole arm translocation associated with monosomy 18p. *Am J Med Genet* **1997**, *71(4)*, 463-6.
7. Binkert, F.; Stranzinger, J.; Schinzel, A. Partial trisomy of chromosome 18 (pter----q12) following a familial 18;21 translocation rcp(18;21)(q12;q11). *Hum Hered* **1990,** *40(2)*, 81-4. doi: 10.1159/000153910.
8. Sebold, C.; Soileau, B.; Heard, P.; Carter, E.; O'Donnell, L.; et al. Whole arm deletions of 18p: medical and developmental effects. *Am J Med Genet A* **2015,** *167A(2)*, 313-23. doi: 10.1002/ajmg.a.36880.
9. Blattner, W.A.; Kistenmacher, M.L.; Tsai, S.; Punnett, H.H.; Giblett, E.R. Clinical manifestations of familial 13;18 translocation. *J Med Genet* **1980**, *17(5)*, 373-9. doi: 10.1136/jmg.17.5.373.
10. Gu, L.; Zhang, H.; Zhu, G. Impact of reciprocal translocation t (18; 21) on male infertility and embryo development: lessons from an oocyte-donating ICSI cycle. *J Assist Reprod Genet* **2011**, *28(7)*, 603-5. doi: 10.1007/s10815-011-9558-0.
11. Chen, C.P.; Chern, S.R.; Lee, C.C.; Chen, L.F.; Chin, D.T.; et al. Prenatal diagnosis of trisomy 18p and distal 21q22.3 deletion. *Prenat Diagn* **2003**, *23(9)*, 758-61. doi: 10.1002/pd.684.
12. Cotton, C.; Cummins, M.; Smith, A. Alternate, adjacent 2 and 3:1 meiotic segregation products from a balanced t(13;18) (q12;q11) carrier. *Clin Genet* **1993**, *44(4)*, 193-5. doi: 10.1111/j.1399-0004.1993.tb03878.x.
